# Retrieving the structure of probabilistic sequences of auditory stimuli from EEG data

**DOI:** 10.1038/s41598-021-83119-x

**Published:** 2021-02-10

**Authors:** Noslen Hernández, Aline Duarte, Guilherme Ost, Ricardo Fraiman, Antonio Galves, Claudia D. Vargas

**Affiliations:** 1 Instituto de Matemática e Estatística, Universidade de São Paulo, São Paulo, Brazil; 2 Instituto de Matemática, Universidade Federal do Rio de Janeiro, Rio de Janeiro, Brazil; 3 Centro de Matemática, Universidad de la República, Montevideo, Uruguay; 4 Instituto de Biofísica Carlos Chagas Filho, Universidade Federal do Rio de Janeiro, Rio de Janeiro, Brazil

**Keywords:** Learning algorithms, Sensory processing

## Abstract

Using a new probabilistic approach we model the relationship between sequences of auditory stimuli generated by stochastic chains and the electroencephalographic (EEG) data acquired while 19 participants were exposed to those stimuli. The structure of the chains generating the stimuli are characterized by rooted and labeled trees whose leaves, henceforth called *contexts*, represent the sequences of past stimuli governing the choice of the next stimulus. A classical conjecture claims that the brain assigns probabilistic models to samples of stimuli. If this is true, then the context tree generating the sequence of stimuli should be encoded in the brain activity. Using an innovative statistical procedure we show that this context tree can effectively be extracted from the EEG data, thus giving support to the classical conjecture.

## Introduction

It has long been conjectured that the brain learns statistical regularities from sequences of stimuli^1^. The ability to extract and represent these regularities over time, often termed as statistical learning^2,3^ plays a crucial role in perception and decision-making^4–6^.

Electrophysiological evidence of statistical learning has been often assessed by identifying signal differences for standard and unexpected stimuli embedded in a sequence^7^, or by looking for fluctuations in the measured signal associated with different surprise levels^8–10^. Much of this research has focused on the identification in the brain activity of signatures of either item frequency or transition probabilities associated to the sequence of stimuli^11–18^.

Here we address the conjecture that the brain learns statistical regularities from sequences of stimuli employing the new probabilistic framework introduced in Duarte et al.^19^. In that framework, participants are exposed to a sequence of auditory stimuli while their electroencephalographic (EEG) activity is measured. The EEG signal is then segmented in the following way. The first EEG segment is the one recorded during the presentation of the first auditory stimulus; the second EEG segment is the one recorded during the presentation of the second auditory stimulus, and so on. Doing so, we now have two coupled sequences: the original sequence of auditory stimuli and the derived sequence of EEG segments recorded during the presentation of the sequence of the successive auditory stimuli. The question is whether the sequence of EEG segments expresses in some way the type of dependence from the past characterizing the sequence of auditory stimuli. A crucial point here is that the length of the relevant sequence of past choices of auditory stimuli governing the transition to the next one does not need to be fixed: it can very well depend on the sequence of past symbols itself. This framework allows to address von Helmholtz’s classical conjecture as follows. Can the information concerning the sequences of past auditory units governing the successive transitions be retrieved from the statistical features of the EEG segments?

This approach is based on a genial intuition presented in Rissanen^20^. He noted that in most sequences of data observed in the real world, each new data unit is chosen in a probabilistic way. This choice is made by taking into account a sequence of past units whose length is not fixed, but changes as a function of the sequence of past units itself. Following Rissanen^20^ we call a *context* the sequence of past units containing the relevant information required to generate the next unit. The set of contexts defines a partition of the set of all possible past sequences and can be represented by a rooted and labeled oriented tree, henceforth called *context tree*. To completely characterize the way the sequence of units is generated, a transition probability governing the choice of the next unit is associated with each context.

Stochastic sequences as described above are called context tree models^20,21^. These models are able to approximate any stationary stochastic chain in an economic way, by using a small number of parameters and thus have been successfully employed to model biological and linguistic phenomena^22–28^.

In Rissanen’s words, von Helmholtz’s classical conjecture can be rephrased as follows. Is it possible to retrieve the context tree generating the sequence of auditory stimuli from the sequence of EEG segments? With this formulation a major challenge is how to identify the statistical features of the EEG segments associated with each context. Duarte et al.^19^ addresses this question from an innovative mathematical point of view. By employing the statistical procedure proposed in Duarte et al.^19^, we show that context trees generating sequence of auditory stimuli can effectively be extracted from EEG data.

## Methods

### Participants

Nineteen individuals (18 right-handed, 9 female, age average 30 and standard deviation 6.8 years) with no reported neurological, neuropsychiatric or auditory disease participated in the study. Participants consisted of three undergraduate and four graduate students as well as 13 health service professionals (physical therapists and psychologists), recruited from the staff of the Laboratory of Neuroscience and Rehabilitation of the Institute of Neurology Deolindo Couto. The menstrual cycle of the female participants was not considered. Participants signed an informed consent term. Research was performed in accordance with the relevant guidelines and regulations and approved by the Research Ethics committee of the Institute of Neurology Deolindo Couto at the Federal University of Rio de Janeiro (Plataforma Brasil process number 22047613.2.0000.5261).

### Stimuli and task

The auditory stimuli consisted of strong beats, weak beats, and silent units presented at stimulus onset asynchrony of 450 ms using the Presentation software, version 16.5, Sound card: SoundMAX HD Audio (https://www.neurobs.com/). Strong and weak beats consisted of hand claps (spectral frequency range 0.2–15 KHz and maximal duration 200 ms each) recorded through a microphone. These auditory stimuli were then digitized and sharp cut to a duration of 450 ms using the software Audacity, version 2.0.5.0 (https://www.audacityteam.org/).

Participants sat in a comfortable chair in a dimly illuminated room. No task was asked for the participants to perform. They were informed neither about the intent of the study, nor about the existence of statistical regularities in the sequence of stimuli. They were only instructed to close their eyes and listen attentively to the sequences of auditory stimuli. Sounds were presented binaurally via headphones at around 60 dB SPL. The loudness of the stimuli was individually regulated before the experiment start by asking each participant to adjust the beat sounds to a comfortable level.

The manner by which the sequences of stimuli were generated is presented in the next sub-section (Structure of the sequences of stimuli). Samples of the employed sequences of stimuli can be found in the “Supplementary Audio” online.

### Structure of the sequences of stimuli

At this point, it is convenient to introduce a few formal definitions. Let *A* be a finite set. Given two integers *m*, *n* with $m\le n$, the string $(u_m,\ldots , u_n)$ of symbols in *A* will be denoted $u^n_m$; its *length* is $\ell (u^n_m)=n-m+1$. We will also use the shorthand notation *u* to denote the sequence $u^n_m$.

Given two sequences *u* and *v* of elements of *A*, *uv* denotes the sequence of length $\ell (u)+\ell (v)$ obtained by concatenating *u* and *v*. The sequence *u* is said to be a *suffix* of *v* if there exists a sequence *s* satisfying $v=su$. When $v\ne u$ we say that *u* is a *proper suffix* of *v*.

A finite set $\tau$ of sequences of elements of *A* is a *context tree* if it satisfies the following conditions.

1. *Suffix property* No $w\in \tau$ is a proper suffix of another element $u \in \tau$.
2. *Irreducibility* No sequence belonging to $\tau$ can be replaced by a proper suffix without violating the suffix property.

The elements of $\tau$, i.e., the *leaves* of the tree, are called *contexts*.

The *height* of the tree $\tau$ is defined as $\ell (\tau )=\max \{\ell (w){\text{: }}\, w \in \tau \}.$ For any sequence *u* with $\ell (u)\ge \ell (\tau )$, we write $c_{\tau }(u)$ to denote the only context in $\tau$ which is a suffix of *u*.

Let $\tau$ be a context tree and $p=\{p(\cdot \mid w){\text{: }} w\in \tau \}$ be a family of probability measures on *A* indexed by the contexts belonging to $\tau$. The pair $(\tau ,p)$ is called a *probabilistic context tree* on *A*.

A stochastic chain $(X_n{\text{: }} n \ge 0)$ taking values in *A* is called a *context tree model* compatible with $(\tau ,p)$ if

1. $\tau$ defines a partition of the set of all possible sequences of past symbols generated by the chain;
2. For any $n\ge \ell (\tau )$ and any finite sequence $x^{-1}_{-n}\in A^{n}$ such that $P\big (X^{n-1}_{0}=x^{-1}_{-n}\big )>0$, we have
  1 $$\begin{aligned} P\left( X_n=a\mid X^{n-1}_{0}=x^{-1}_{-n}\right) =p\left( a\mid c_{\tau }\left( x^{-1}_{-n}\right) \right) \ \ \text{ for } \text{ all } \ a\in A; \end{aligned}$$
3. No proper suffix of $c_{\tau }\left( x^{-1}_{-n}\right)$ satisfies condition 2.

Given a probabilistic context tree $(\tau ,p)$, the algorithm to generate a context tree model can be summarized as follows:

- Start the sample choosing, in an arbitrary way, any context with length equal to $\ell (\tau )$;
- at each subsequent step *n*, identify the context $c_{\tau }\big (X^{n-1}_{0}\big )$ corresponding to the past sequence $X_0,\ldots ,X_{n-1}$;
- choose the symbol $X_n$ using the transition probability $p\left( \cdot |c_{\tau }\left( X^{n-1}_{0}\right) \right)$.

For more details on context tree models we refer the reader to the founding papers by Rissanen^20^ and by Bühlmann and Wyner^22^ and to the survey by Galves and Löcherbach^21^.

**Figure 1:**
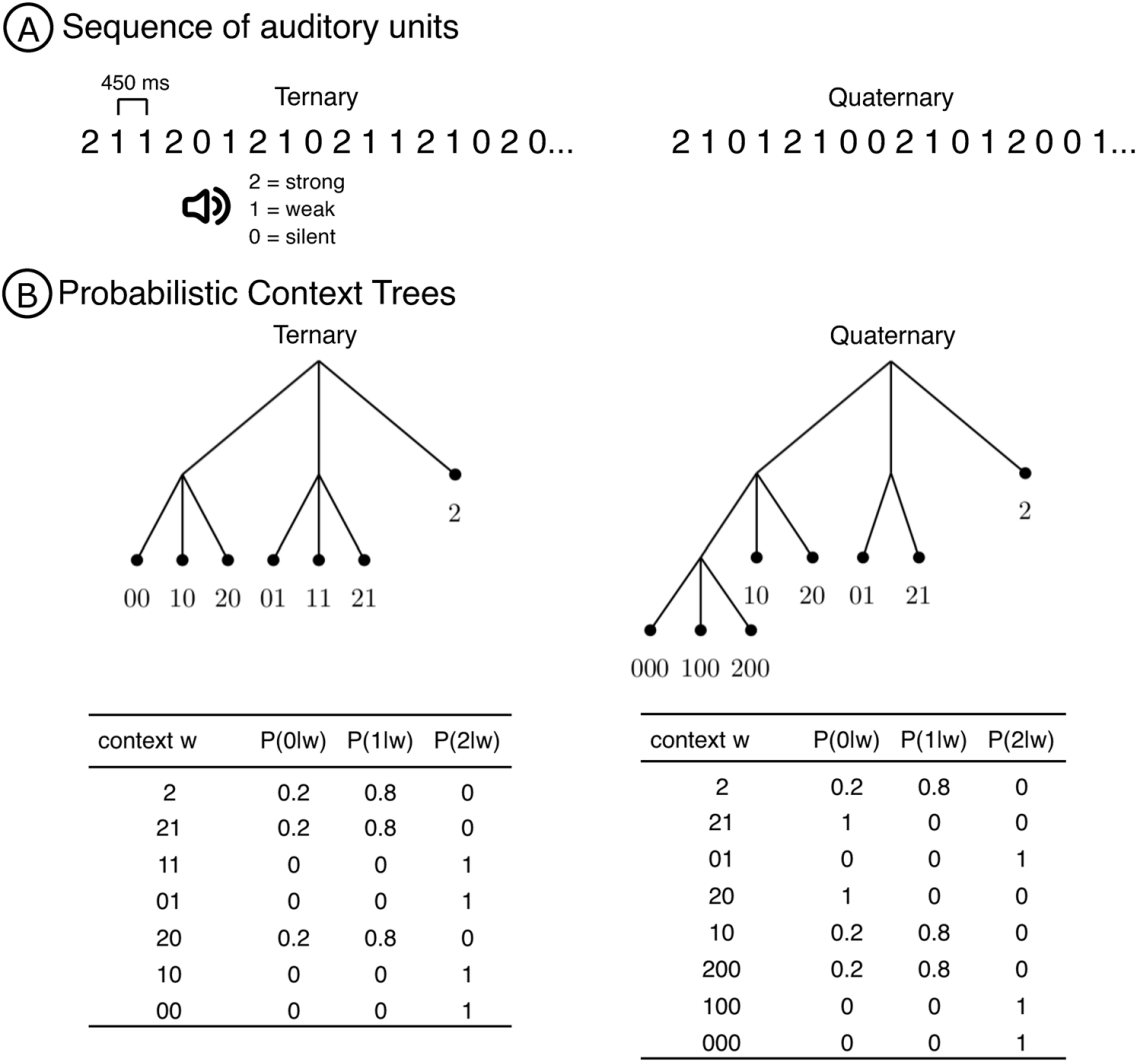
Experimental design. (**A**) Participants were exposed to sequences of auditory stimuli of three different types: strong beats, weak beats, and silent units, presented at every 450 ms. (**B**) Ternary and Quaternary context trees together with their associated family of transition probabilities. The transition probability associated to a context is used to choose the type of auditory stimulus appearing after the ocurrence of that context in the sequence of stimuli.

We can now describe in a precise way the context tree models used to generate the sequences of stimuli. Take $A=\{0,1,2\}$, where 0 represents a silent unit, 1 represents a weak beat and 2 represents a strong beat (Fig. 1A). In our experiment, we used two different context tree models to generate the sequences of stimuli, namely the *Ternary* and *Quaternary* conditions (Fig. 1B). These two context trees reproduce mathematically two popular rhythms. The Quaternary condition is a simplified version of the Brazilian Samba. The Ternary condition is based on the Waltz structure.

In the Ternary condition, the tree of contexts is $\tau = \{00,10,20,01,11,21,2\}$ and the family of associated probability transitions is such that

- if the sequence of past symbols ends either with context 2, or with context 21, or with context 20, the next symbol will be either 0, with probability 0.2, or 1, with probability 0.8. The symbol 2 is not allowed to occur after contexts 2, 21 or 20;
- if the sequence of past symbols ends with context 11, or with context 10, or with context 01, or with context 00, the next symbol will be 2, with probability 1. The symbols 0 and 1 are not allowed to occur after contexts 11, 10, 01 and 00 (see the left lower table on Fig. 1B).

In the Quaternary condition, the tree of contexts is $\tau = \{000,100, 200, 10, 20,01,21,2\}$ and the family of associated probability transitions is such that

- if the sequence of past symbols ends with context 2, or with context 10, or with context 200, the next symbol will be either 0, with probability 0.2, or 1 with probability 0.8. The symbol 2 is not allowed to occur after contexts 2, 10 or 200;
- if the sequence of past symbols ends with the context 21, or with the context 20, the next symbol will be 0, with probability 1. The symbols 1 and 2 are not allowed to occur after contexts 21 or 20;
- if the sequence of past symbols ends with context 01, or with context 100, or with context 000, the next symbol will be 2, with probability 1. The symbols 0 and 1 are not allowed to occur after contexts 01, 100 or 000 (see the right lower table in Fig. 1B).

Finally we consider sequences of stimuli generated by choosing at each step either 0 or 1 or 2 with equal probabilities 1/3, 1/3 and 1/3 independently of the past choices. This will be called the Independent condition. The goal of introducing this condition was to *shuffle cards* before the participant is exposed to a next sample.

For each condition, sequences of stimuli were generated independently per participant. In this way, each participant was exposed to different sequences of stimuli, all of them, however, being generated by the same context tree model.

### Experimental design and procedures

Each participant was exposed to two 12.5 min blocks of sequences of auditory stimuli. The blocks were separated by a period of time ranging from 5 to 10 min, during which no data was acquired. Each 12.5 min block was composed by three 3.5 min sub-blocks. Each sub-block was a concatenation of three 1 min sequences of auditory units generated using either the Ternary, the Independent or the Quaternary trees. Each sequence of auditory units was separated from the next one by a 15 s silent interval and each sub-block with three sequences was separated from the next one by a 1 min silent interval.

For half of the volunteers the starting 12.5 min block contains sub-blocks of the type Ternary, Independent, Quaternary and the second 12.5 min block contains sub-blocks of the type Quaternary, Independent, Ternary. The inverse ordering was used with the other half, to balance possible order effects.

### Data preprocessing

EEG data was acquired at 250 Hz, using a 128 channels system (Geodesic HidroCel GSN 128 EGI, Electrical Geodesic Inc.). The electrode positioned on the vertex (Cz) was used as a reference during the acquisition. The electrode cap was immersed in saline solution (KCl) prior to data collection. During acquisition, the signal was amplified with a nominal gain of 20 times and analogically filtered (Butterworth first order band-pass filter of 0.1–200 Hz; Geodesic EEG System 300, Electrical Geodesic Inc.).

**Figure 2:**
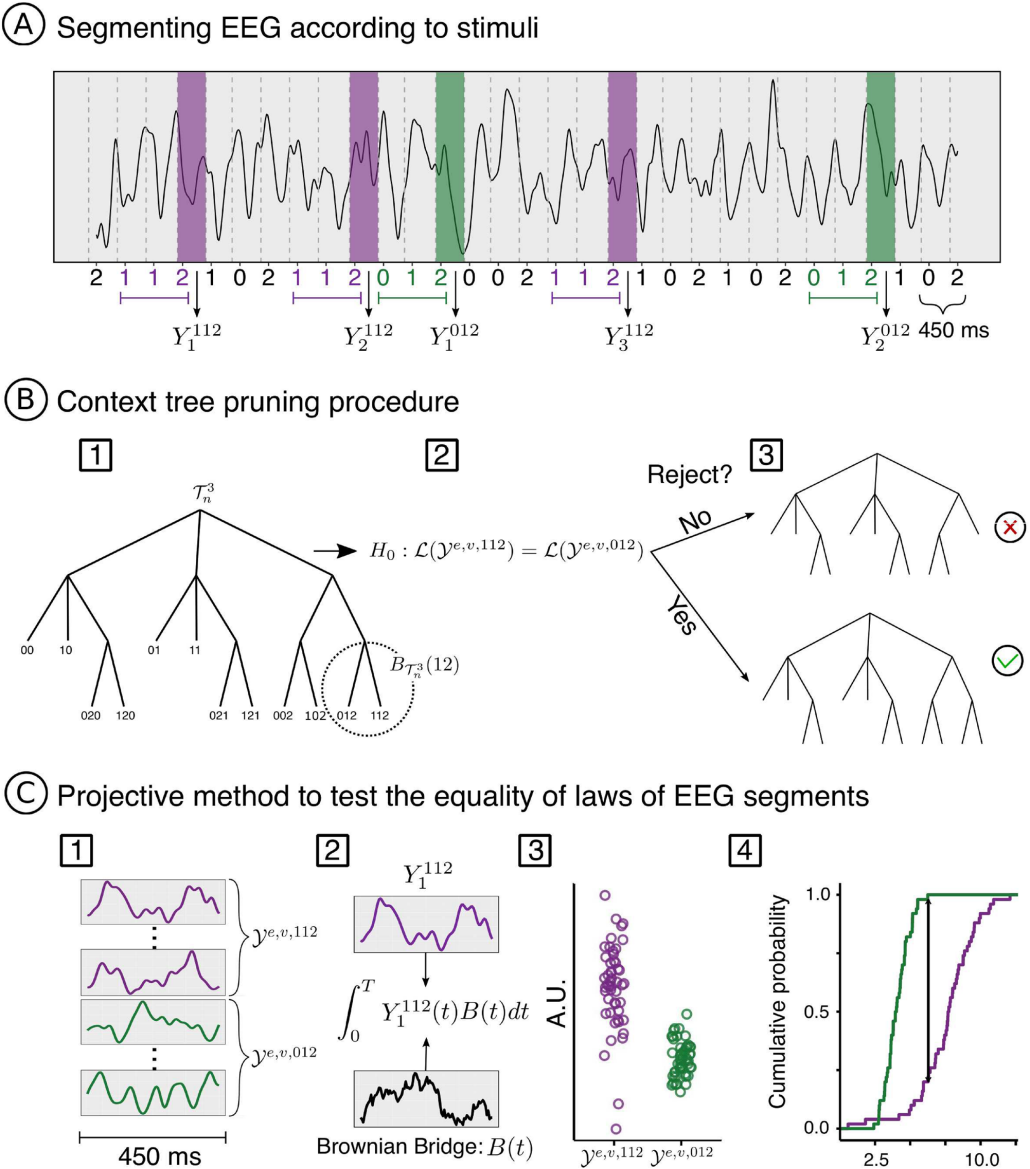
Context tree estimation pipeline. (**A**) EEG signal of one electrode segmented in windows of 450 ms, starting 50 ms before the presentation of each stimulus. (**B**) The pruning procedure is performed by (1) selecting not visited terminal subtrees of the complete tree, (2) testing the null hypothesis of equality of distributions for the sample of EEG segments associated to the leaves of that subtree, and (3) pruning the subtree if the null hypothesis is not rejected for all pairs of leaves in the subtree or keeping the subtree if the null hypothesis is rejected for at least one pair of leaves. (**C**) The test of the null hypothesis for the equality of distributions is performed by (1) selecting EEG segments associated with the pair of strings corresponding to the leaves that will be tested, (2) performing the inner product of the EEG segments with a realization of a Brownian bridge to obtain the projections, (3) having a distribution of the projections, (4) testing the null hypothesis for the equality of distributions for projections using the Kolmogorov–Smirnov test.

The recorded signal was re-referenced to the average reference using NetStation software (Electrical Geodesic Inc), version 4.3.1 (https://www.egi.com/research-division/net-station-eeg-software) and filtered offline with a Butterworth fourth order band-pass filter of 1–30 Hz. EEG signal was then epoched with a peri-stimulus window of $-50$ to 400 ms (Fig. 2A) using EEGLAB^29^ (https://sccn.ucsd.edu/eeglab/index.php) running in MATLAB environment, version R2015a (https://www.mathworks.com/products/matlab.html). Baseline correction was performed employing the EEG signal collected from $-50$ to 0 ms before the auditory stimuli onset. The window of interest (from $-50$ to 400 ms relative to the onset of the auditory stimulus) was chosen so as to include the duration of the auditory stimulus processing, spanning up to around 400 ms (see, for instance, Naatanen et al.^30^; Garrido et al.^12^).

### Statistical data analysis

The statistical procedure employed to estimate a context tree for each participant and for each electrode was introduced in Duarte et al.^19^, to which we refer the reader for a complete description of the method including the proofs of the consistency result. This procedure is depicted in Fig. 2.

Let *V* be the set of all participants and *E* the set of all electrodes considered in the analysis. In our experiment the sets *V* has 19 elements and *E* is a set of 18 electrodes of the international 10–20 system

$$\begin{aligned} E =\{\text{ FP1 }, \text{ FP2 }, \text{ F7 }, \text{ F3 }, \text{ Fz }, \text{ F4 }, \text{ F8 }, \text{ T7 }, \text{ C3 }, \text{ C4 }, \text{ T8 }, \text{ T7 }, \text{ P3 }, \text{ Pz }, \text{ P4 }, \text{ P8 }, \text{ O1 }, \text{ O2 }\}. \end{aligned}$$

For each participant $v \in V$ and each electrode $e \in E$, let $X_1^{v},\ldots ,X_n^{v}$ be the sequence of stimuli the participant is exposed to, and $Y_n^{e,v}$ be the segment of EEG data recorded during the exposure to the auditory stimulus $X_n^v$. The sequence $(X_1^v,Y_1^{e,v}),\ldots ,(X_n^v, Y_n^{e,v})$ will be used to estimate the context tree ${\hat{\tau }}_n^{e,v}$. This is done by a pruning procedure starting with an admissible context tree of maximal height *k* for the sequence of stimuli $X_1^v,\ldots , X_n^v$, denoted by ${\mathcal {T}}_n^k$, where *k* is a suitable positive integer.

Following Duarte et al.^19^, an admissible context tree of maximal height *k* is any irreducible tree of height *k* in which all the contexts are finite sequences of symbols in the set $\{0,1,2\}$ that effectively occur in the sequence of stimuli. As an illustration, Fig. 2B, step 1, shows an admissible context tree of maximal height 3 for the Ternary condition.

Before summarizing the pruning procedure, it is convenient to introduce a new definition. A *subtree* in a tree $\tau$ induced by the sequence *u* is defined as the set $B_{\tau }(u) = \{w \in \tau : u { \text{ is } \text{ a } \text{ suffix } \text{ of } } w\}$. The subtree $B_{\tau }(u)$ is called *terminal* if for all $w \in B_{\tau }(u)$ it holds that $w = au$ for some $a \in A$ (see Fig. 2B, step 1).

Given a tree $\tau$ and $w = w_{-k}^{-1}\in \tau$, let ${\mathcal {Y}}^{e,v,w}$ be the set of EEG segments recorded during the occurrence of the auditory unit $w_{-1}$, when it appears after the units $w_{-k}^{-2}$ (in Fig. 2A, EEG segments associated to the leaves 012 and 112 are marked in green and purple, respectively).

The pruning procedure can be informally described as follows.

1. Compute ${\mathcal {T}}_n^k$ which is the admissible context tree of maximal height *k*, for the sequence of stimuli $X_1^v,\ldots , X_n^v$.
2. We start the pruning procedure from the admissible context tree $\tau = {\mathcal {T}}^k_n$.
3. For each terminal subtree $B_{\tau }(u)$ on $\tau$ and for all pairs of leaves $au, bu \in B_{\tau }(u)$ with $a,b \in A$, test the null hypothesis that the distribution of the EEG chunks ${\mathcal {Y}}^{e,v,au}$ and ${\mathcal {Y}}^{e,v,bu}$ associated to the leaves *au*, *bu* is equal (Fig. 2B, step 2).
  - If the null hypothesis is not rejected for any pair of leaves $au, bu \in B_{\tau }(u)$, we conclude that the occurrence of the symbol *a* or *b* before the sequence *u* does not affect the distribution of EEG chunks and we prune the terminal subtree $B_{\tau }(u)$. Pruning the subtree $B_{\tau }(u)$ means updating the set $\tau$ by eliminating from it the elements in $B_{\tau }(u)$ and adding to it the sequence *u* (Fig. 2B, step 3). Formally, $\tau = \tau \setminus B_{\tau }(u) \cup \{u\}.$
  - If the null hypothesis is rejected for at least one pair of leaves *au* and *bu*, we conclude that the distribution of EEG chunks depends on the entire sequence *au* and *bu* and we keep the terminal subtree $B_{\tau }(u)$ in $\tau$ (Fig. 2B, step 3).
4. Repeat step 2 until all terminal subtrees have been tested.
5. Call ${\hat{\tau }}_n^{e,v}$ the tree constituted by the sequences in $\tau$ when the pruning procedure ends.

Figure 2B exemplifies the pruning of the terminal subtree $\{012, 112\}$. At this step, we want to test if the distribution of the EEG responses to the string 2 preceded by the strings 01 and the EEG responses to the string 2 preceded by the string 11 are different. On one hand, if the distribution of the EEG responses are identical this means the last symbol in the past (i.e., 0 in the string 012 and 1 in the string 112) does not influence the EEG responses. In this case we discard the strings 012 and 112 in the tree, and replace them by the string 12. On the other hand, if the distribution of the EEG responses are not identical this means the last symbol in the past (i.e., 0 in the string 012 and 1 in the string 112) does influence the EEG responses. In this case the strings 012 and 112 are not pruned, becoming contexts of the tree. Notice that with the analysis of this subtree, we can not conclude that the EEG responses to token 2 are not influenced by two symbols backwards in the past. To do so, we need also to analyze the terminal subtree $\{002, 102\}$.

To test the equality of distributions for two samples of EEG segments, Duarte et al.^19^ use the projective method proposed in Cuesta-Albertos et al.^31^. This method takes a function and transforms it into a real number (i.e., its projection in a Brownian Bridge). The theorem presented in Cuesta-Albertos et al.^31^ guarantees that if we reject the null hypothesis that the distribution of the projections corresponding to two samples of functions are equal, we can also reject the null hypothesis that the two samples of functions have the same distribution in the functional space. To obtain the projections corresponding to the EEG segments, we perform an inner product of the EEG function with a realization of a Brownian Bridge. The equality of distribution for the resulting samples of real numbers is tested using the Kolmogorov–Smirnov test, with alpha at 0.05 (Fig. 2C). We use the same realization of the Brownian Bridge to project all EEG segments. To guarantee the stability of the estimated context trees, we perform the projection 5000 times using different Brownians, which implies performing 5000 hypothesis tests. We only reject the null hypothesis that the distributions are the same if more than 276 null hypothesis are rejected. This threshold was derived according to a binomial distribution where the probability of success corresponds to the probability of rejecting an individual test assuming the null hypothesis is true, which is 0.05.

After estimating one context tree per electrode per participant, ${\hat{\tau }}_n^{e,v}$, we ended up on each electrode *e* with a set of context trees $T_n^e= \{{\hat{\tau }}_n^{e,v}, v\in V\}$ corresponding to the context trees estimated for all participants *V* in that electrode. We summarized this set of trees in a unique tree ${\tilde{\tau }}^e$ called *mode context tree*, defined as follows.

The sequence *w* is a context in ${\tilde{\tau }}_n^e$ if the following conditions hold:

(i) $${\Sigma _{v \in V}} {1}_{\left\{ w \in {\hat{\tau }}_n^{e,v}\right\} }>  \underset{u{\text{:}}w\,{\mathrm{proper\,suffix\,of }}\,u} \max {\Sigma _{v \in V}} {1}_{\left\{ u \in {\hat{\tau }}_n^{e,v}\right\} }$$
(ii) Condition (i) is not fulfilled for any sequence *u* which is a proper suffix of *w*.

**Figure 3:**
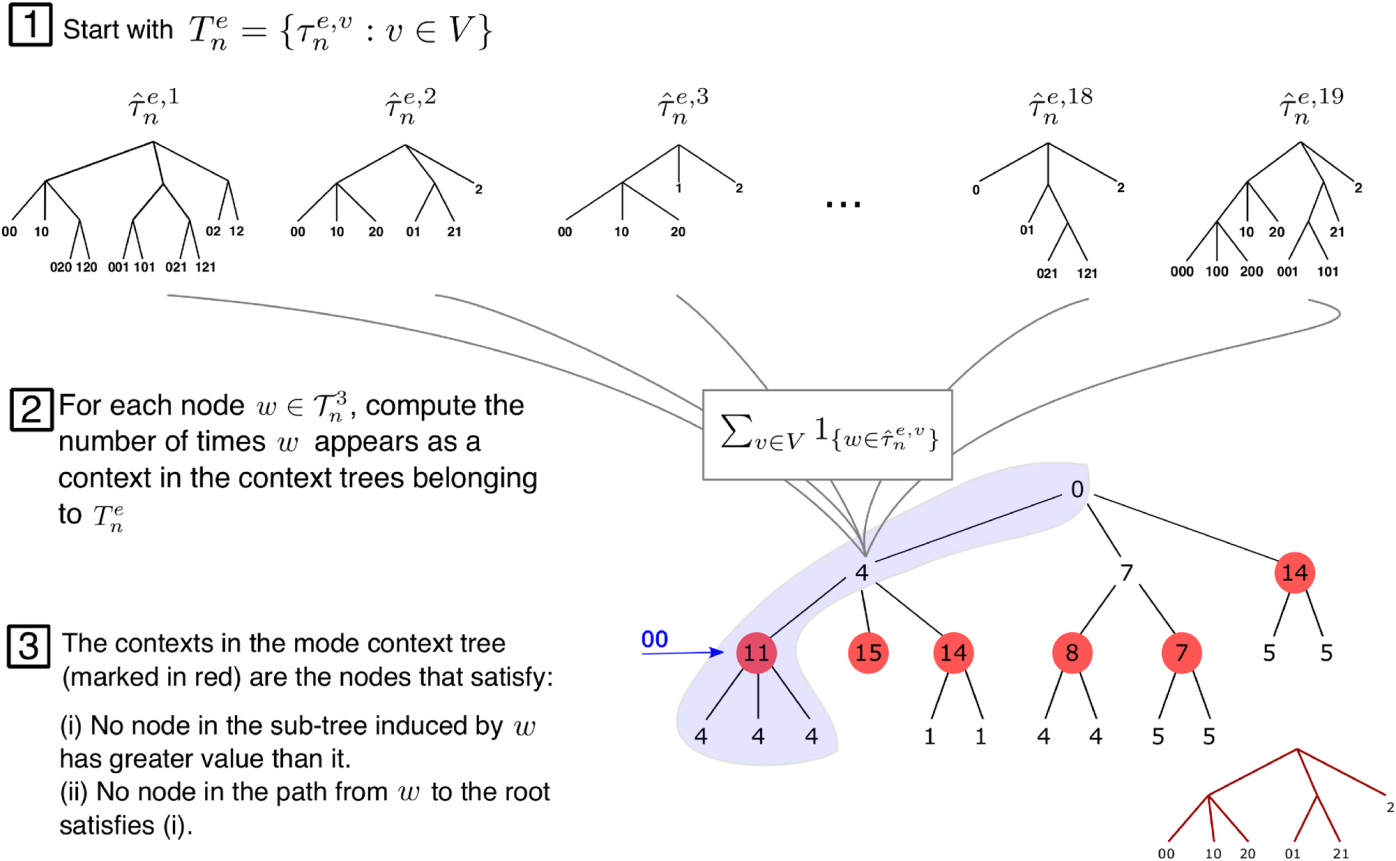
Mode context tree estimation. Start with the set $T_n^e$ which contains the context tree estimated from the data collected at electrode *e* across participants. Then, for each node $w \in {\mathcal {T}}_n^3$, with ${\mathcal {T}}_n^3$ the admissible context tree of maximal height 3, we compute $\Sigma _{v \in V} \mathbb {1}_{\{w \in {\hat{\tau }}_n^{e,v}\}}$ which is the number of times *w* appears as a context in the context trees retrieved from data collected at electrode *e* across participants. In the Figure these contexts are indicated in red. The contexts of the mode context tree are the nodes that satisfy (i) no node in the subtree induced by *w* has greater value than it; (ii) no node in the path from *w* to the root satisfies (i). For instance, the node 00 appears 11 times, while the nodes 000, 100 and 200 which are in the subtree induced by 00 appear four times each. Besides, the ancestor nodes 0 and the root appear four times and zero time, respectively. Therefore, 00 appears as a context in the mode context tree.

In words, a sequence *w* is a context in the mode context tree corresponding to an electrode *e*, when *w* is more often a context in $T^e$ than any sequence for which *w* is a suffix. Furthermore, no suffix of *w* can satisfy the above condition. Thus, the leaves (i.e. contexts) of the mode context tree are those sequences that were identified as contexts more often across participants. Figure 3 exemplifies the computation of the mode context tree.

## Results

### Retrieving context trees from EEG data

We estimated a context tree per electrode from the EEG data collected while the participants were exposed to sequences of auditory stimuli generated by the Ternary and Quaternary conditions. For each condition and each electrode, the mode context tree summarizes the results obtained across participants.

**Figure 4:**
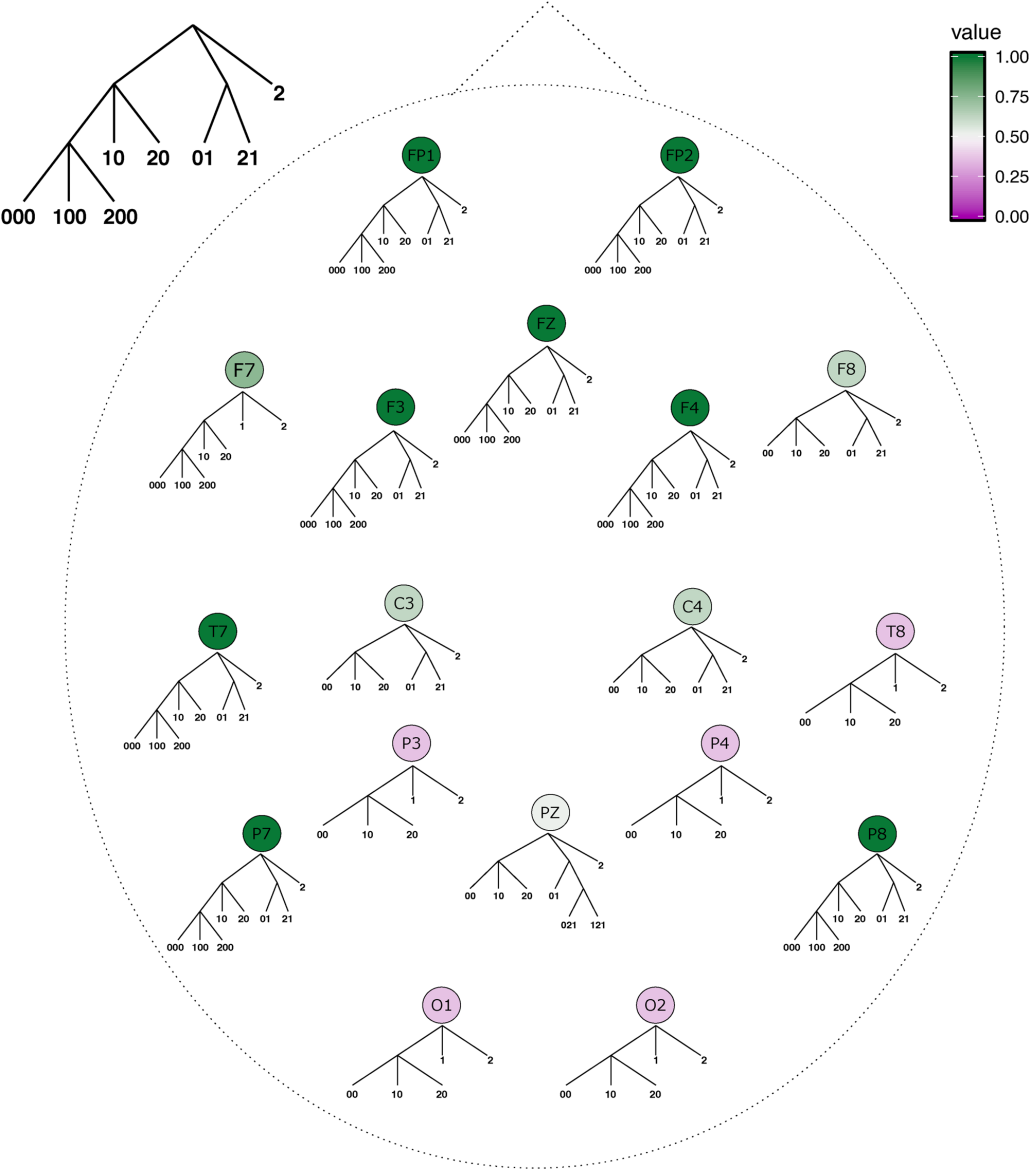
Mode context trees across electrodes for the Quaternary condition. For each electrode, the mode context tree represents the sequences that were more often estimated as contexts across participants for sequences of stimuli generated by the Quaternary context tree. The color scale indicates the similarity of a mode context tree ${\hat{\tau }}^e$ with the Quaternary context tree $\tau _{qua}$ computed as $s({\hat{\tau }}^e, \tau _{qua}) = \Sigma _{w \in \tau _{qua}} (1/8) \mathbb {1}_{\{w \in {\hat{\tau }}^e\}}$. The closer to one the similarity value, the more similar the two trees are.

The results obtained in the Quaternary condition are plotted in Fig. 4. In five frontal (FP1, FP2, FZ, F3, and F4), two parietal (P7 and P8) and one temporal (T7) electrode, the mode context tree exactly matches the tree that generated the sequence of stimuli, namely $\tau = \{000, 100, 200, 10, 20, 01, 21, 2\}$.

In addition, the estimated mode context tree only misidentified 1 as a context in F7 and 00 as a context in F8, C3 and C4. In PZ, the mode context tree misidentified 00, 021 and 121 as contexts. Finally, for T8, P3, P4, O1 and O2, besides the lack of the third order contexts 000,100 and 200, the symbol 1 is misidentified as a context.

Summarizing, contexts 2, 10 and 20 were perfectly identified in all the 18 electrodes; contexts 01 and 21 were well identified in 11 out of 18 electrodes and the third order contexts 000,100 and 200 were identified in 9 out of 18 electrodes.

**Figure 5:**
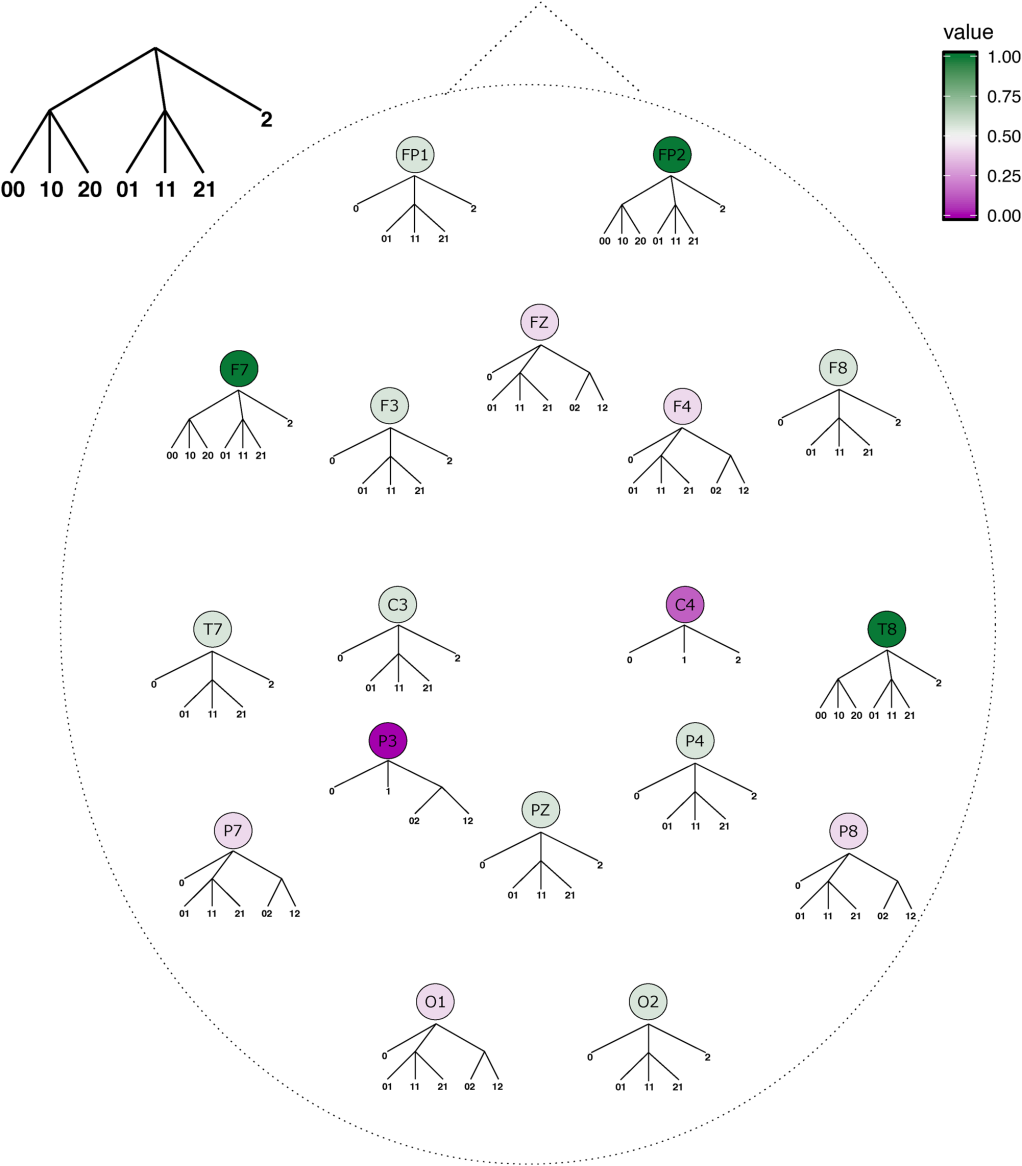
Mode context trees across electrodes for the Ternary condition. For each electrode, the mode context tree represents the sequences that were more often estimated as contexts across participants for sequences of stimuli generated by the Ternary context tree. The color scale indicates the similarity of a mode context tree ${\hat{\tau }}^e$ with the Ternary context tree $\tau _{ter}$ computed as $s({\hat{\tau }}^e, \tau _{ter}) = \Sigma _{w \in \tau _{ter}} (1/7) \mathbb {1}_{\{w \in {\hat{\tau }}^e\}}$. The closer to one the similarity value, the more similar the two trees are.

Results for the Ternary condition are plotted in Fig. 5. For this condition, the mode context tree obtained for electrodes FP2, F7 and T8 was exactly the one that generated the stimuli, namely $\tau = \{00,10,20,01,11,21,2\}$. Moreover, 12 out of 18 electrodes (FP2, FP1, F7, F3, F8,T8, C4, C3, T7, PZ, P4, O2) had 2 as a context. Also, 16 out of 18 electrodes (FP1, FP2, F7, F3, FZ, F4, F8, T7, C3, T8, PZ, P4, P7, P8, 01, 02) contained the subtree $\{01,11,21 \}$. Surprisingly, the second order dependencies preceding the silent unit in the subtree $\{00,10,20\}$ were present only in 3 out of 18 mode context trees. Finally, inexistent second order dependencies appeared in electrodes FZ, F4, P7, P8 and O1.

### Interindividual variability

To evaluate the interindividual variability, for each electrode we calculated the distance between the mode context tree and the context trees retrieved across participants, as in Balding et al.^32^. Informally speaking, the distance between two context trees is the weighted average of the boolean variables associated to each candidate node of the tree. For each node the boolean variable assumes the value 1 if the node belongs to one of the two context trees, but not to both, otherwise it assumes the value 0. We refer the reader to Balding et al.^32^ for a formal definition.

**Figure 6:**
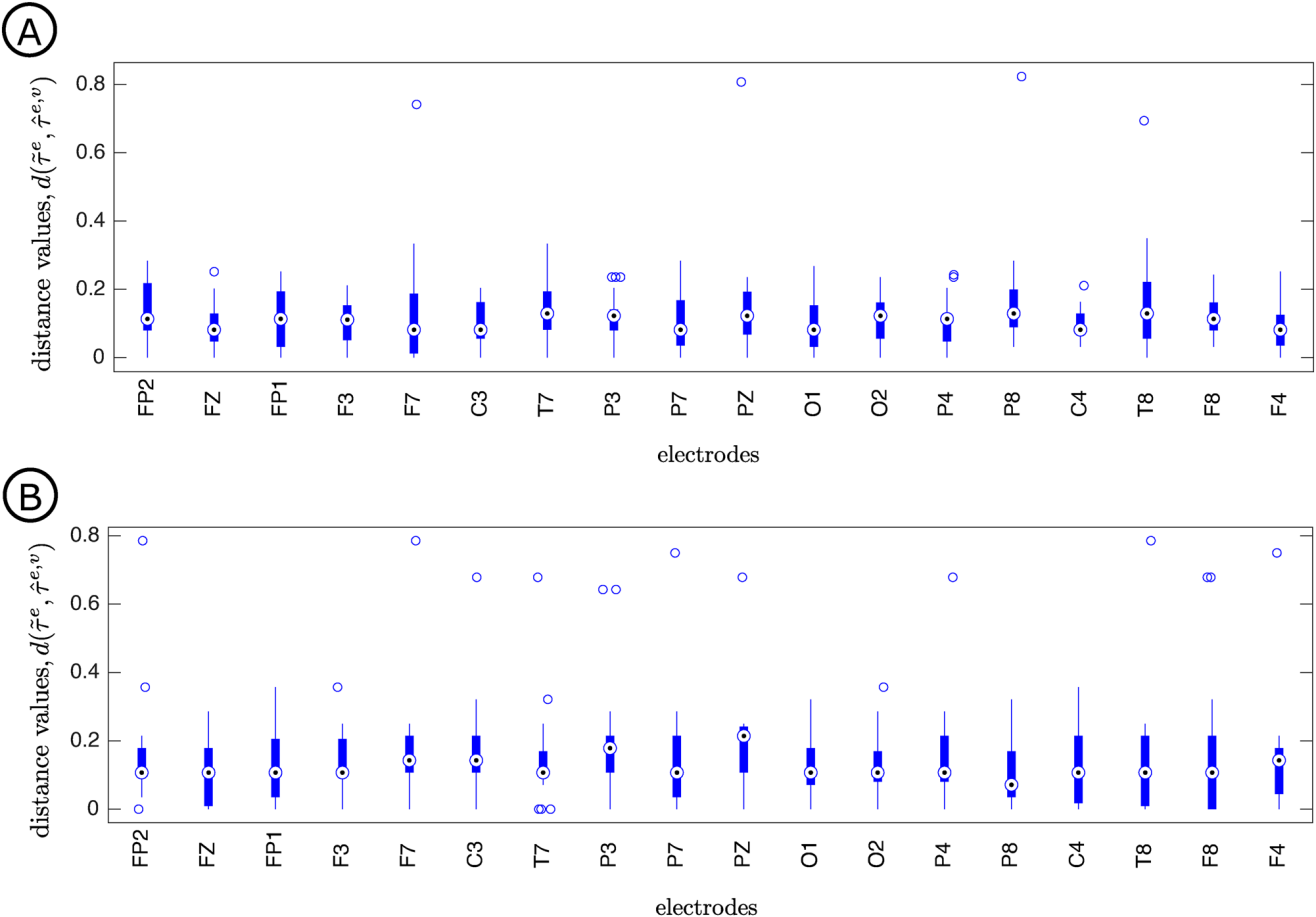
Boxplots depict the distance values between the mode context tree ${\tilde{\tau }}^e$ and the context tree retrieved per participant ${\hat{\tau }}^{e,v}, v \in V$ at a given electrode *e* for the Quaternary (**A**) and Ternary (**B**) conditions.

Figure 6 depicts the boxplots of the set of distance values between the mode context tree and the context trees retrieved per participant at a given electrode for the Quaternary (A) and the Ternary (B) conditions.

It is evident from Fig. 6 that the individual context trees retrieved per participant at a given electrode concentrate around the mode context tree. Moreover, both the median of the distances and the interquartile range are small. The fact that for each electrode the distances between the mode context tree and the retrieved context trees across participants are concentrated in a small interval around the median value indicates a relatively low interdindividual variability.

The complete sets of context trees estimated per participant and electrode for each condition are presented in Figs. S5–S40 in the “Supplementary material”. Furthermore, the number of times each finite sequence of stimuli was estimated as a context across participants for each electrode on each condition are depicted in Figs. S1–S4 in the “Supplementary material”.

### Likelihood of obtaining the correct context tree by chance

To evaluate how likely it is to obtain the results above just by chance we did a simulation experiment. This experiment assumes that the retrieved context trees have been chosen in a purely random and uniform way. In the Quaternary condition the pruning procedure could return a context tree among 51 possible candidates. The candidates are the context trees that could be produced by successively pruning subtrees in all possible cases, starting from the admissible context tree of maximal height 3. In the Ternary condition the set of possible candidates contains 46 context trees.

For each condition, we simulate this situation by randomly choosing sets of 19 trees uniformly in the set of possible candidates context trees. We repeat this procedure 10,000 times. For each set of 19 randomly chosen context trees we compute the mode context tree.

**Figure 7:**
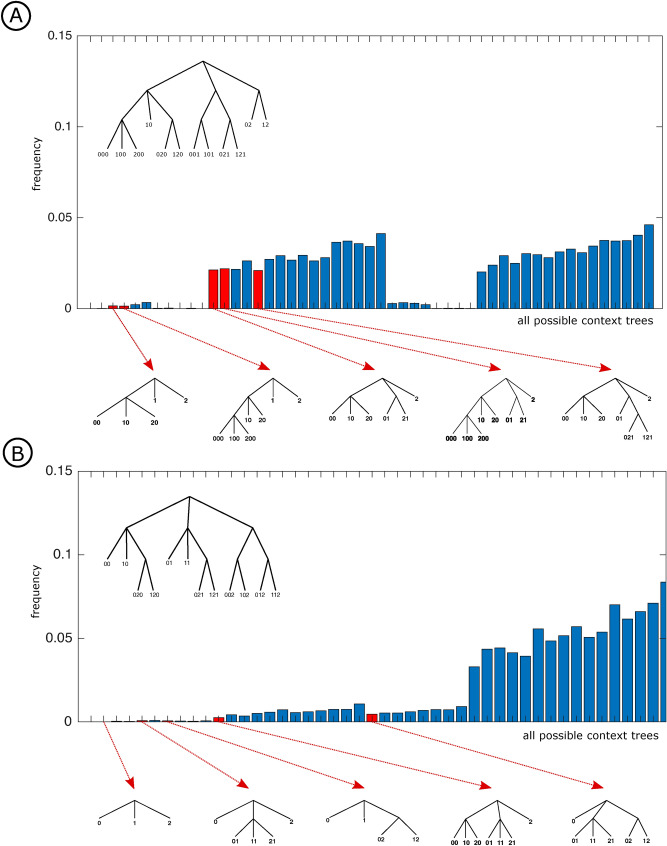
Empirical distribution of the mode context trees obtained by randomly choosing sets of 19 trees uniformly in the set of possible candidate context trees both for the Quaternary (**A**) and the Ternary (**B**) conditions. For each set of 19 randomly chosen context trees we compute the mode context tree. This procedure is repeated 10,000 times. In the histogram, the trees appearing from the experimental data analysis are indicated. The corresponding admissible context trees of maximal height 3 are depicted at the top of each plot.

Figure 7 presents the empirical distribution of the mode context trees obtained by means of the random choice described above for both conditions. In the Quaternary condition (Fig. 7A), the resulting empirical distribution assigns a probability close to 0.02 to the Quaternary tree that generated the sequence of stimuli.

If the result obtained with the EEG data collected in the Quaternary condition was produced by chance, as it was the case in the simulation, its likelihood could be easily computed as follows. As indicated in Fig. 4, the Quaternary tree appears as mode context tree across participants in eight electrodes, namely FP1, FP2, FZ, F3, F4, T7, P7 and P8. Assuming that the data collected at each one of the 18 electrodes returns the Quaternary tree by chance and independently of the other electrodes with probability 0.02, then the probability that the Quaternary tree appears as mode context tree in exactly eight out from 18 electrodes can be computed by a Binomial distribution with parameters 18 and 0.02. The probability that a Binomial distribution with parameters 18 and 0.02 takes the value 8 is equal to ${18 \atopwithdelims ()8} * (0.02)^8 * (0.98)^{10} \approx 1/10^{9}$, which is overwhelmingly small. Using the Binomial we can also compute the probability that the Quaternary tree appears at least eight times among the 18 context tree modes. This probability is of the order of $1/10^{9}$. These results show that obtaining the Quaternary tree in at least eight out of 18 context tree modes can hardly be explained by pure chance. Similar results can be obtained in the Ternary case.

## Discussion

In the present study, we employed a new statistical framework^19^ to retrieve from EEG signals the structure of the algorithm governing the production of a sequence of auditory stimuli. For each electrode and condition the set of context trees retrieved accross participants was summarized in the mode context tree associated to each electrode and condition.

Both for the Quaternary and the Ternary conditions, the context trees generating the sequences of stimuli were completely retrieved from the EEG signals recorded in certain electrodes. For other electrodes, however, the retrieved mode context trees did not match exactly the ones that generated the sequences of stimuli. Notwithstanding, some contexts were estimated quite consistently both for the Quaternary and the Ternary conditions. For instance, context 2 appeared in all mode context trees for the Quaternary condition, and in 12 out of 18 mode context trees for the Ternary condition. In addition, the subtree $\{01, 21\}$ appeared in the mode context trees obtained in 11 out of 18 electrodes for the Quaternary condition, and the subtree $\{01, 11,21\}$ appeared in 16 out of 18 mode context trees for the Ternary condition. Finally, the subtree $\{000, 100, 200\}$ was present in 9 out of 18 mode context trees in the Quaternary condition and the subtree $\{00,10,20\}$ was retrieved only in 3 out of 18 mode context trees in the Ternary condition.

Previous MEG/EEG and fMRI studies had shown that brain signals can be associated with transition probabilities of first order^8,9,33,34^. Furthermore, studies using serial reaction time tasks and artificial grammar learning had proven that humans can learn stimuli sequences that contain higher-order temporal dependencies^35–38^. Our results show that it was possible to retrieve second ($\{01,21\}$) and third ($\{000, 100, 200\}$) order temporal dependencies from EEG signals in the Quaternary condition. Likewise, second order dependencies ($\{01, 11, 21\}$) could be retrieved in the Ternary condition. Furthermore, our approach allowed to go beyond those previous reports by identifying the context trees characterizing the learned temporal dependencies.

Statistical learning paradigms have mostly relied on the detection of the Mismatch Negativity, consisting in an electrophysiological response induced by a deviant stimulus within a sequence of otherwise regular stimuli^7,39,40^. Accordingly, in our experiment the retrieving of some subtrees could be explained by the presence of a Mismatch Negativity response. For instance, in the Quaternary condition, consider the subtree $\{000,100,200\}$. The silent unit 0 appearing as third element in each of these strings appears as a surprise in two of the strings and is expected to occur in the third string. In effect, the silent unit 0 was unexpected after either 00 or 10 and was expected to occur after the string 20. Therefore, the distinction between the EEG segments collected while the silent unit 0 appears after 00 versus its occurrence after 20 is a typical situation in which the Mismatch Negativity approach would be successful in distinguishing the statistical features of the two EEG signals. The same occurs when we compare the EEG segments collected while the silent unit 0 appears after 10 versus its occurrence after 20. So these are situations in which the Mismatch Negativity effect contributes to the context identification task.

Using the context tree approach, however, we went beyond the identification of a Mismatch Negativity response. This is the case of the frequently retrieved subtree $\{01,21\}$ in the Quarternary condition. In effect, the weak beat 1 appearing as second element in the two strings is expected to occur with high probability in both cases. It occurs with high probability after the context 2 and it occurs also with high probability either after 200 or after 10. In these cases, the identification of a difference in the EEG collected while the weak beat unit is presented either after context 2 or after contexts 200 and 10 can not be explained by the mismatch negativity effect. The successful retrieving of a context in these cases attests that the volunteer is learning the structure of the sequence of stimuli beyond the surprise level. This strongly suggests that the participants succeeded in identifying the context tree generating the stimuli sequence. This context tree identification would allow predicting the upcoming stimulus^41–43^.

It is interesting to observe that in some situations the Mismatch Negativity effect can mask the differences between the laws of the EEG segments. For instance, in the Ternary condition, our model predicts that the subtree $\{00, 10, 20\}$ should appear in the retrieved context tree. It turns out that this only occurs in 3 out of 18 electrodes. This is surprising since to well predict the next symbol it is crucial to distinguish between 00 or 10 in one side, and 20 in the other side. After each of the first two strings a strong beat always appear, while after 20 a weak beat will occur with high probability and a silent unit will occur with small probability. How can we explain that we did not succeed in identifying this distinction in the EEG data more often? This could be due to the fact that in this case the silent unit represented by the symbol 0 appearing at the end of the pair is always the consequence of erasing the most probable weak beat represented by the symbol 1. This is a typical situation of mismatch negativity in the EEG signal triggered by the omission response^44^. Maybe this explains the difficulty in distinguishing the probability distributions generating the EEG segments associated to the leaves of this subtree.

Regarding the spatial distribution of the retrieved mode context trees in the scalp, we found that those corresponding exactly to the tree that generated the sequence of stimuli were mostly obtained in the frontal region. Correctly identifying all the contexts at a particular scalp position could indicate that the temporal dependencies characterizing the sequence of stimuli are preferably encoded by the underlying brain networks. This is consistent with fMRI studies showing that the prefrontal cortex is involved in learning context-based statistics^34,45^.

The results also show some variability regarding the context trees estimated across the scalp. One could suppose that several putative context tree models are being generated across the scalp and used to make predictions. Keeping multiple models is advantageous in volatile environments, where transition probabilities or even temporal dependencies change across time^45,46^.

A common issue in the analysis of functional data such as EEG segments is the need of some type of normalization to compare quantities like the maximum amplitude or the variance of the signals collected from different participants. The context tree approach intrinsically normalizes the data per participant and electrode. This is done by extracting the essential features concerning equality of the probability distributions. These features are then represented by the retrieved context tree. Data from different participants are only put together at the end through the mode context trees. Summarizing the context trees retrieved per electrode and participant using the mode context tree is a novelty introduced in the present paper.

The goal of the present article was to find experimental evidence that it is possible to retrieve from EEG data the features of the probabilistic algorithm governing the successive stimuli presented to a volunteer. One could suppose that a large number of volunteers would be required to achieve this goal. Our results on the interindividual variability suggest however a quite homogeneous performance across participants and conditions.

A main concern refers to the minimum size of the sequence of stimuli required to consistently retrieve the context tree per participant, electrode and condition. First of all, it has been rigorously proven^19^ that our statistical procedure is consistent. A simulation study^19^ indicated that for sequences of auditory stimuli longer than 200 units, the proportion of correctly identified context trees exceeded 0.95. In our experimental protocol, the sequences of auditory stimuli have almost 800 units, which should be sufficiently large to well identify the context trees from the EEG data. Obviously, a major difference between the simulation study and the EEG data analysis is the much smaller variability of the diffusion processes used in the simulation study as proxies of the EEG segments^19^. The intrinsic variability of the EEG data implies in a higher variance of the results and pushes towards using larger sequences of auditory stimuli. Increasing the length of the sequences of stimuli has however the drawback of longer experimental sessions^47^. The homogeneity of the results obtained across participants and contexts suggests that we achieved a good balance between the push towards longer sequences of stimuli and the limitations imposed by increasing the length of the experimental session. As a final note, subjects are supposedly learning the statistics of the different conditions as they undertake them. Thus the derivation of the context trees from EEG should improve over time. Unfortunately, the employed total sample length was much too short to allow the identification of a learning curve.

Humans are great at learning structured sequences of stimuli such as those found in language and music^7^. Our approach allowed retrieving from the collected EEG signals the higher-order temporal dependencies present both in the Ternary and the Quaternary conditions. Our results indicate that the statistical regularities learned from a relatively short exposure to these structured sequences of auditory stimuli become fully encrypted in brain signals. Interestingly, the context trees governing the generation of the sequences of the stimuli were retrieved mostly from EEG signals acquired in frontal electrodes, suggesting that these regions are more likely responsible for encoding higher-order temporal dependencies characterizing the context trees driving the sequences of auditory stimuli.

## Supplementary information

Supplementary Figures.

Supplementary Information 1.

Supplementary Information 2.

Supplementary Information 3.

## Data Availability

The EEG data and the source code used in the statistical analyses can be downloaded from https://neuromat.numec.prp.usp.br/neuromatdb/EEGretrieving/.
