# Supplementary material for: Retrieving the structure of probabilistic sequences of auditory stimuli from EEG data
Source: Sci Rep. 2021 Feb 10;11:3520. doi: 10.1038/s41598-021-83119-x (PMC7875997; doi:10.1038/s41598-021-83119-x)
Supplement: Supplementary file 1 — Supplementary Figures. [file 41598_2021_83119_MOESM1_ESM.pdf]

Retrieving the structure of probabilistic sequences of auditory stimuli from EEG data

Noslen Hernández[1], Aline Duarte[1], Guilherme Ost[2], Ricardo Fraiman[3], Antonio Galves[1], and Claudia D. Vargas[2,*]

[1]Instituto de Matemática e Estatística, Universidade de São Paulo, Brazil
[2]Instituto de Matemática, Universidade Federal do Rio de Janeiro, Brazil
[3]Centro de Matemática, Universidad de la República, Uruguay
[4]Instituto de Biofísica Carlos Chagas Filho, Universidade Federal do Rio de Janeiro, Brazil
[*]cdvargas@biof.ufrj.br

Supplementary Figures

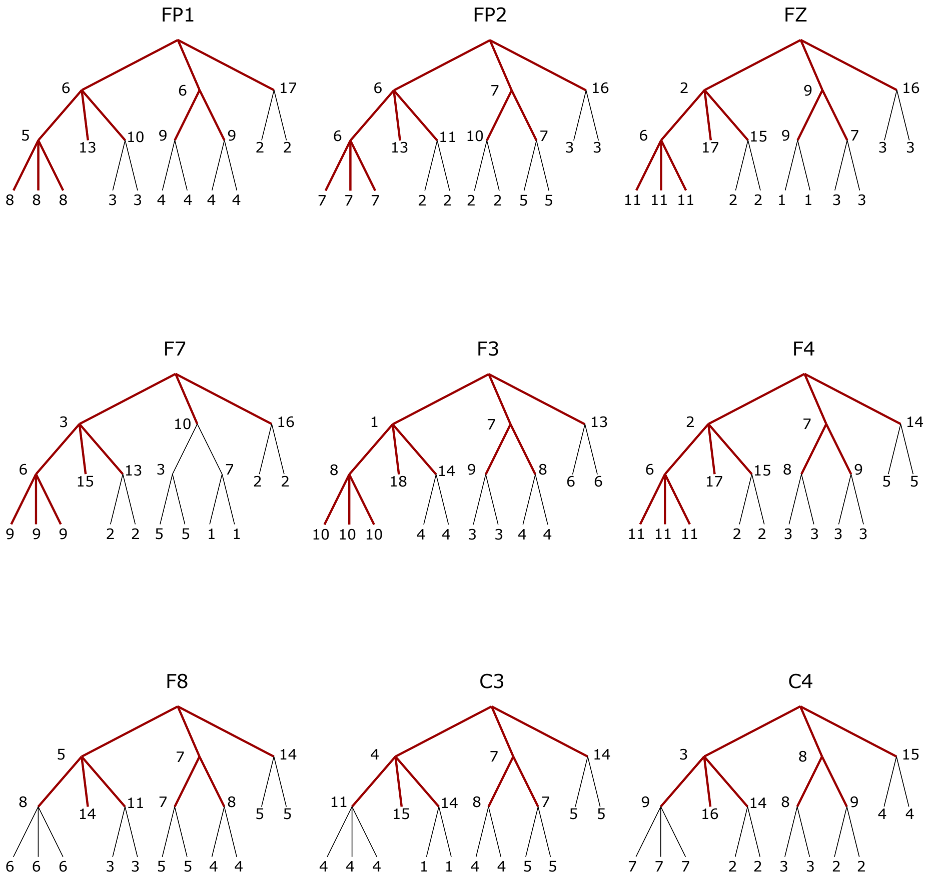

Supplementary Figure S1. Complete context tree of height 3 for the Quaternary condition showing the number of time each node was identified as a context across participants for electrodes FP1, FP2, Fz, F7, F3, F4, F8, C3 and C4. These frequencies are used to obtain the mode context tree which is highlighted in red.

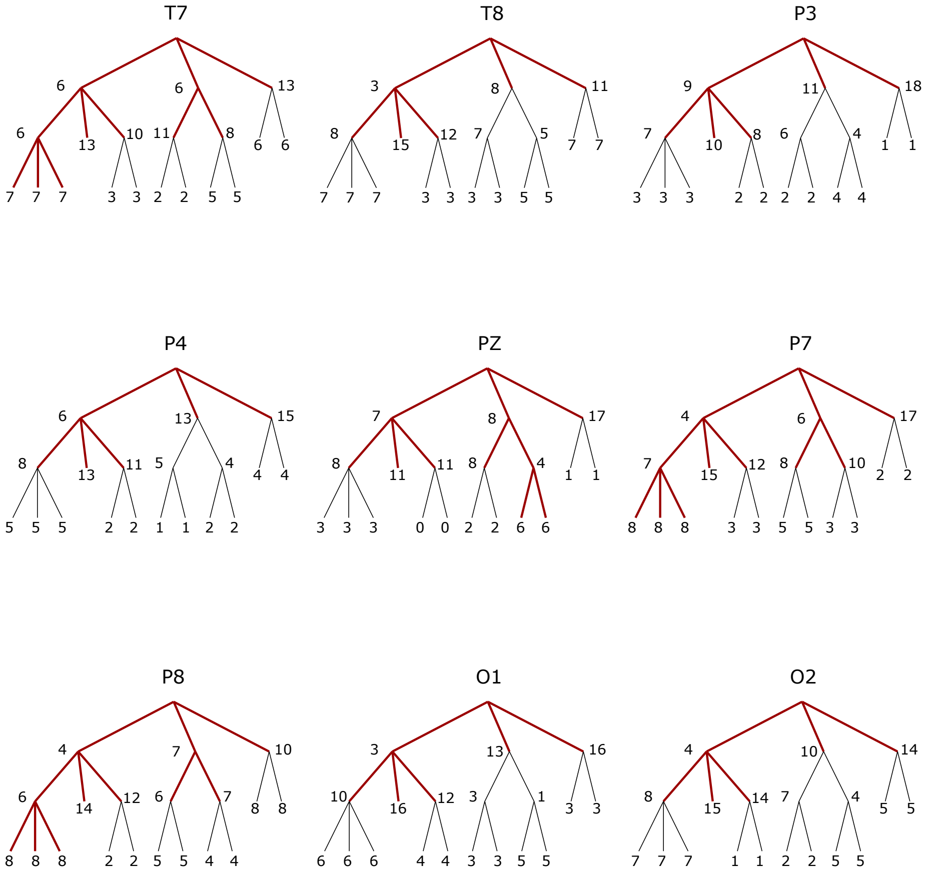

Supplementary Figure S2. Complete context tree of height 3 for the Quaternary condition showing the number of time each node was identified as a context across participants for electrodes T7, T8, P3, P4, Pz, P7, P8, O1 and O2. These frequencies are used to obtain the mode context tree which is highlighted in red.

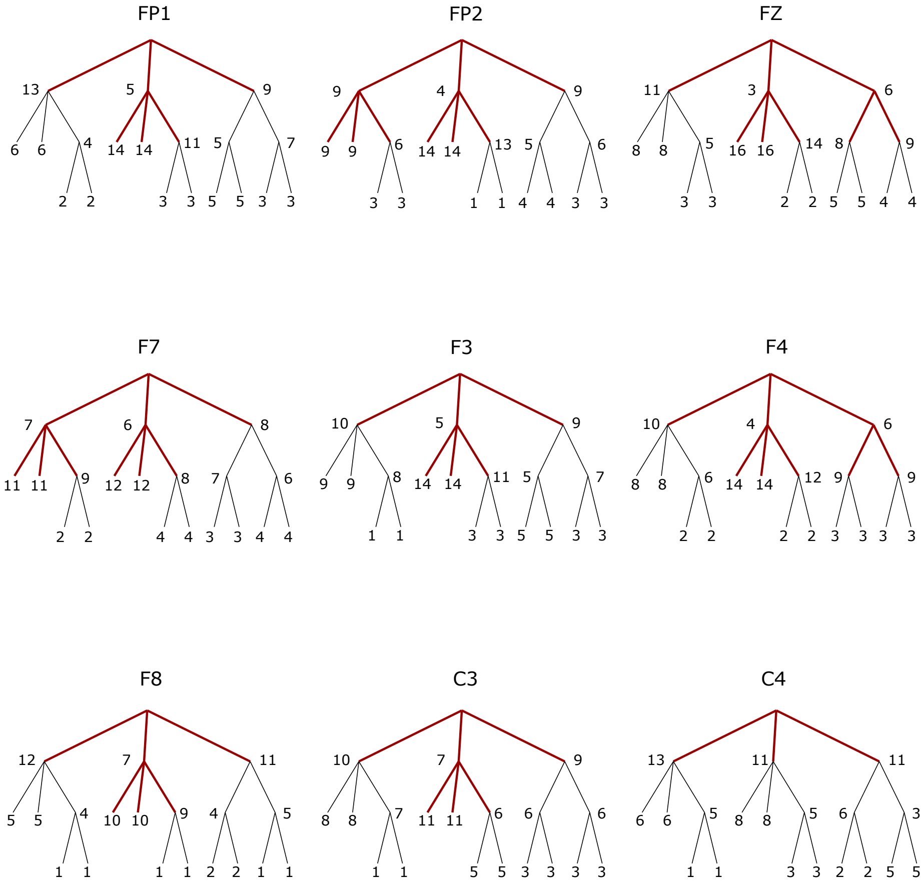

Supplementary Figure S3. Complete context tree of height 3 for the Ternary condition showing the number of time each node was identified as a context across participants for electrodes FP1, FP2, Fz, F7, F3, F4, F8, C3 and C4. These frequencies are used to obtain the mode context tree which is highlighted in red.

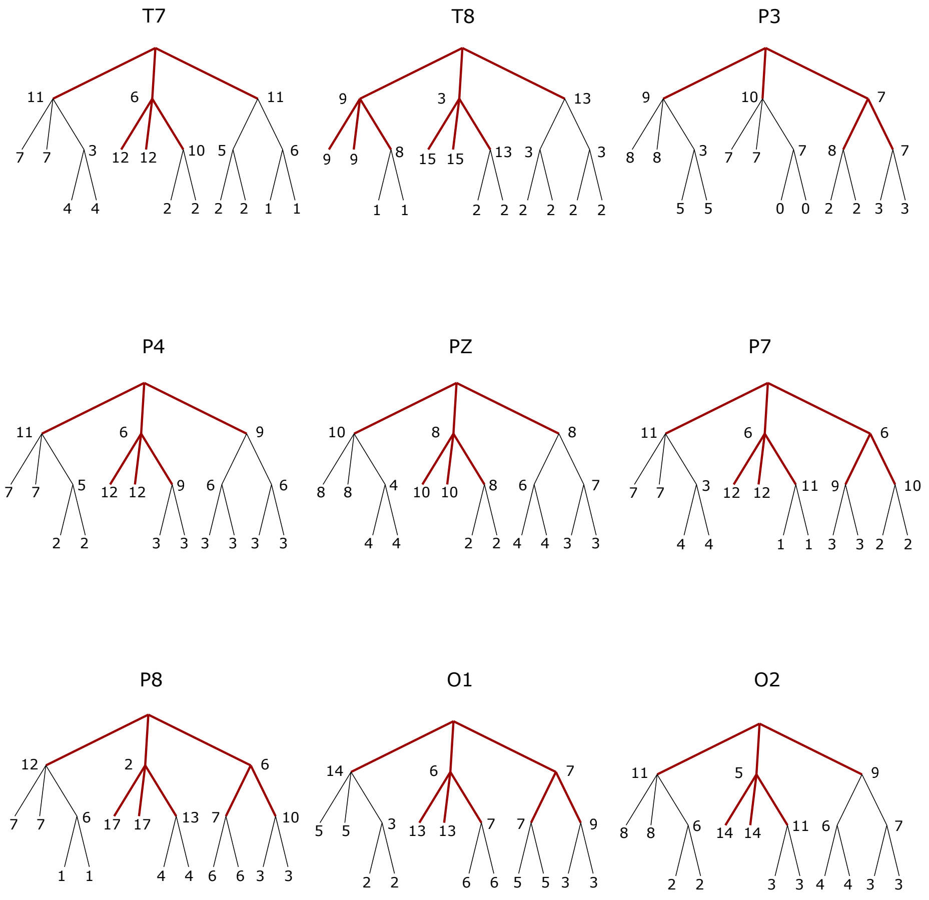

Supplementary Figure S4. Complete context tree of height 3 for the Ternary condition showing the number of time each node was identified as a context across participants for electrodes T7, T8, P3, P4, Pz, P7, P8, O1 and O2. These frequencies are used to obtain the mode context tree which is highlighted in red.

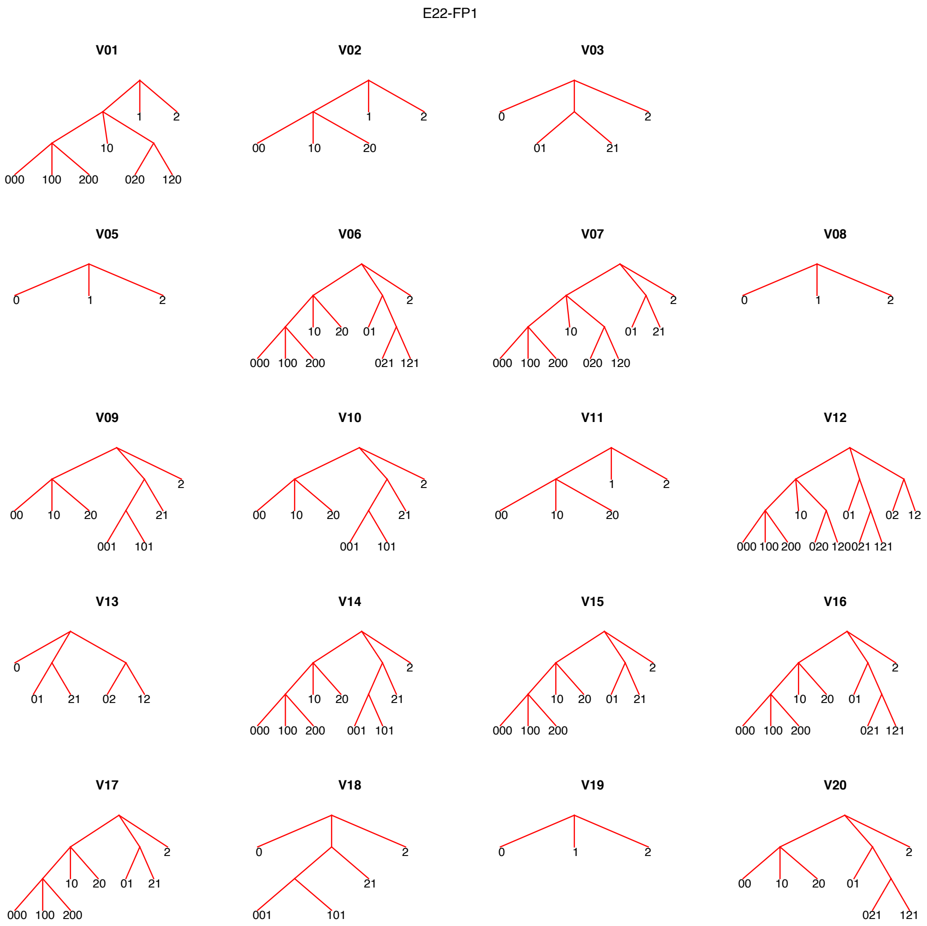

Supplementary Figure S5. Context tree estimated for each participant on electrode FP1 for the Quaternary condition.

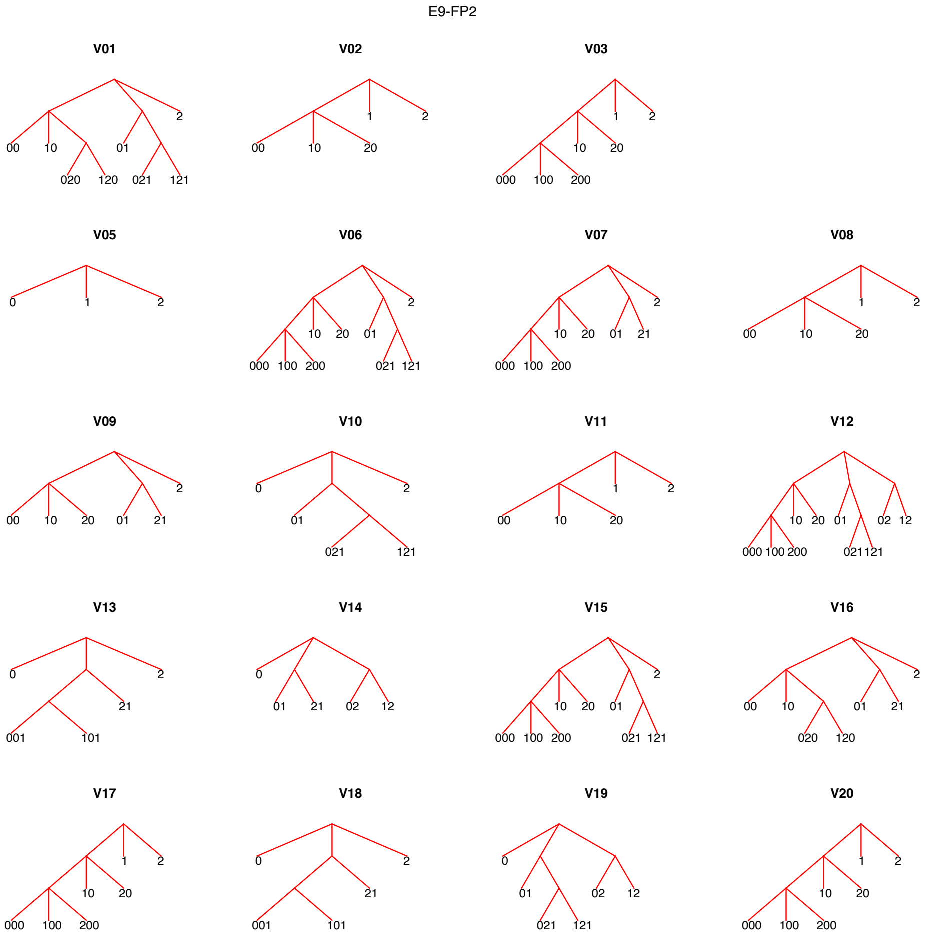

Supplementary Figure S6. Context tree estimated for each participant on electrode FP2 for the Quaternary condition.

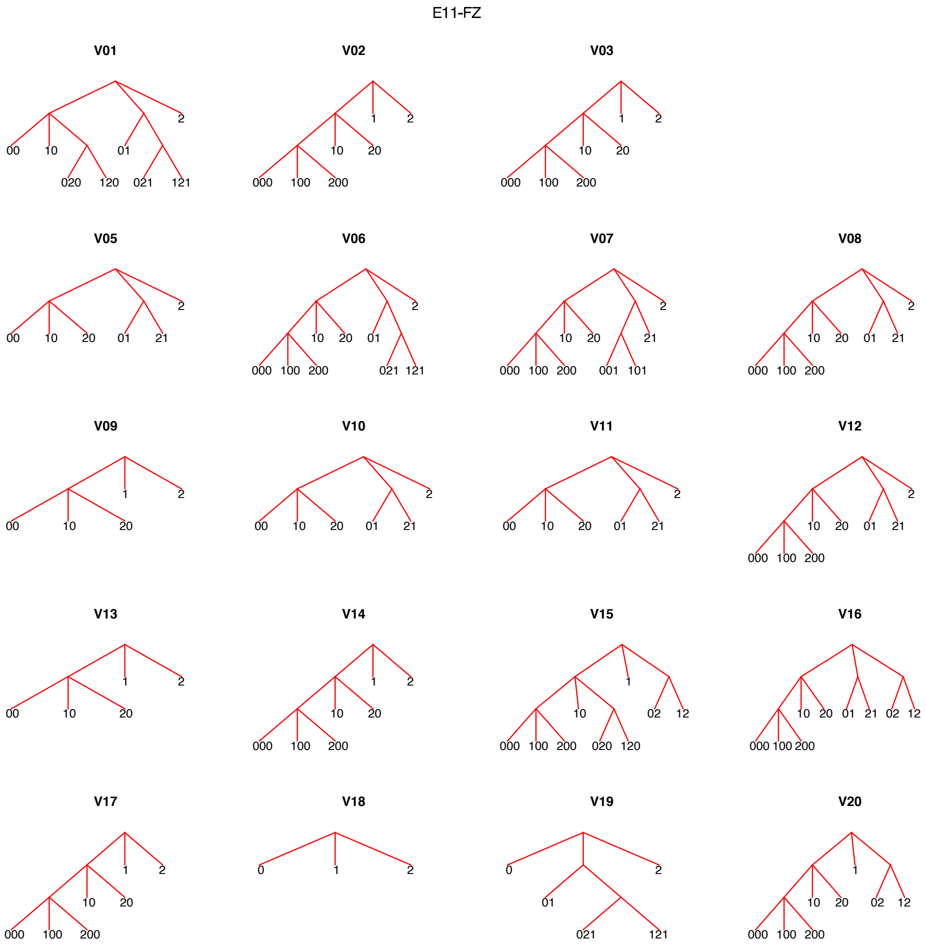

Supplementary Figure S7. Context tree estimated for each participant on electrode FZ for the Quaternary condition.

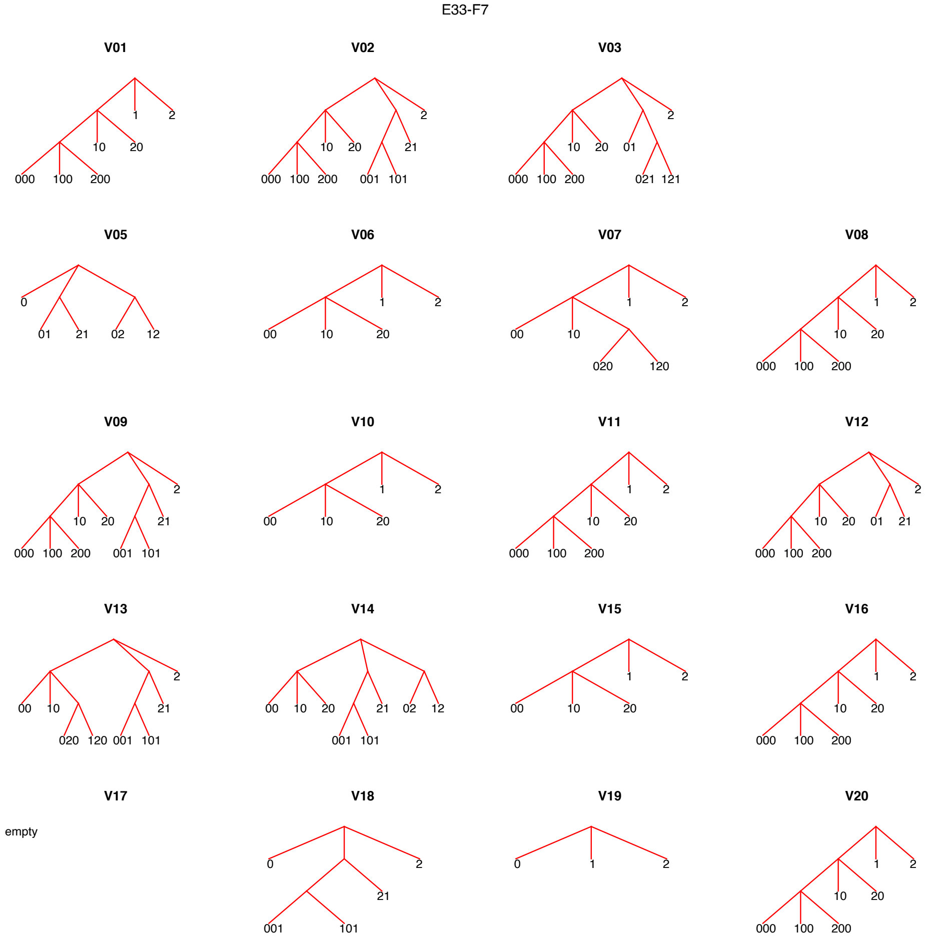

Supplementary Figure S8. Context tree estimated for each participant on electrode F7 for the Quaternary condition.

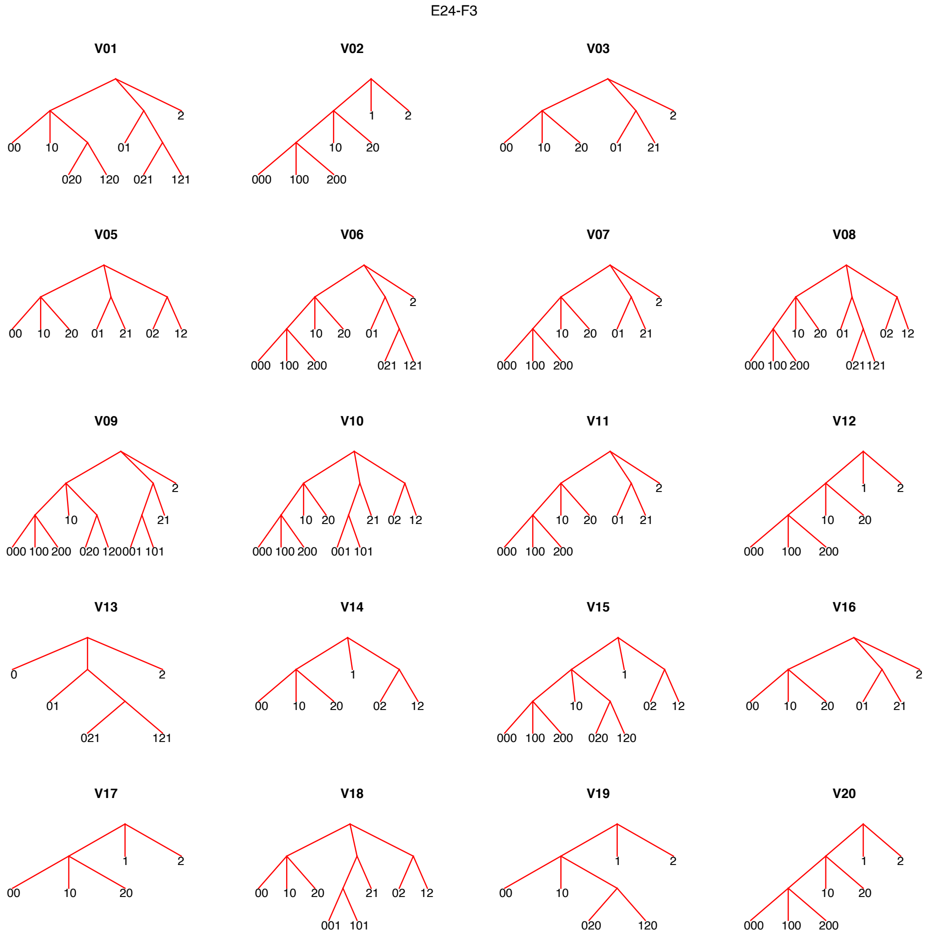

Supplementary Figure S9. Context tree estimated for each participant on electrode F3 for the Quaternary condition.

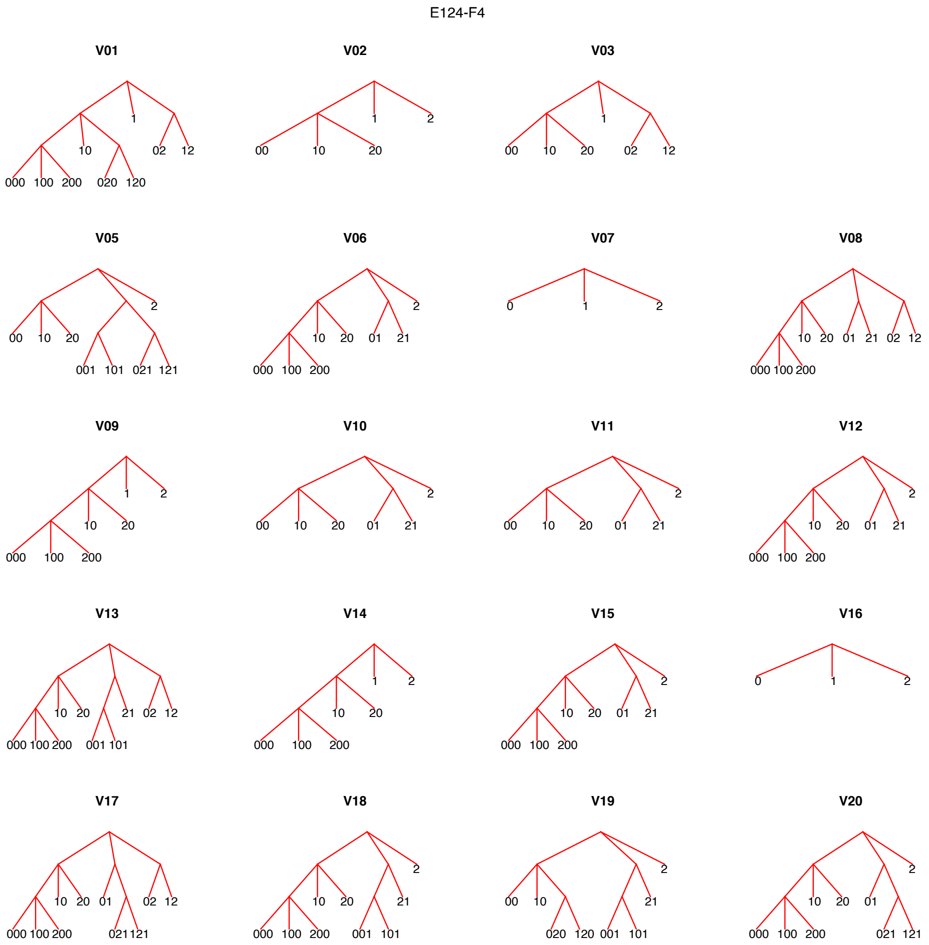

Supplementary Figure S10. Context tree estimated for each participant on electrode F4 for the Quaternary condition.

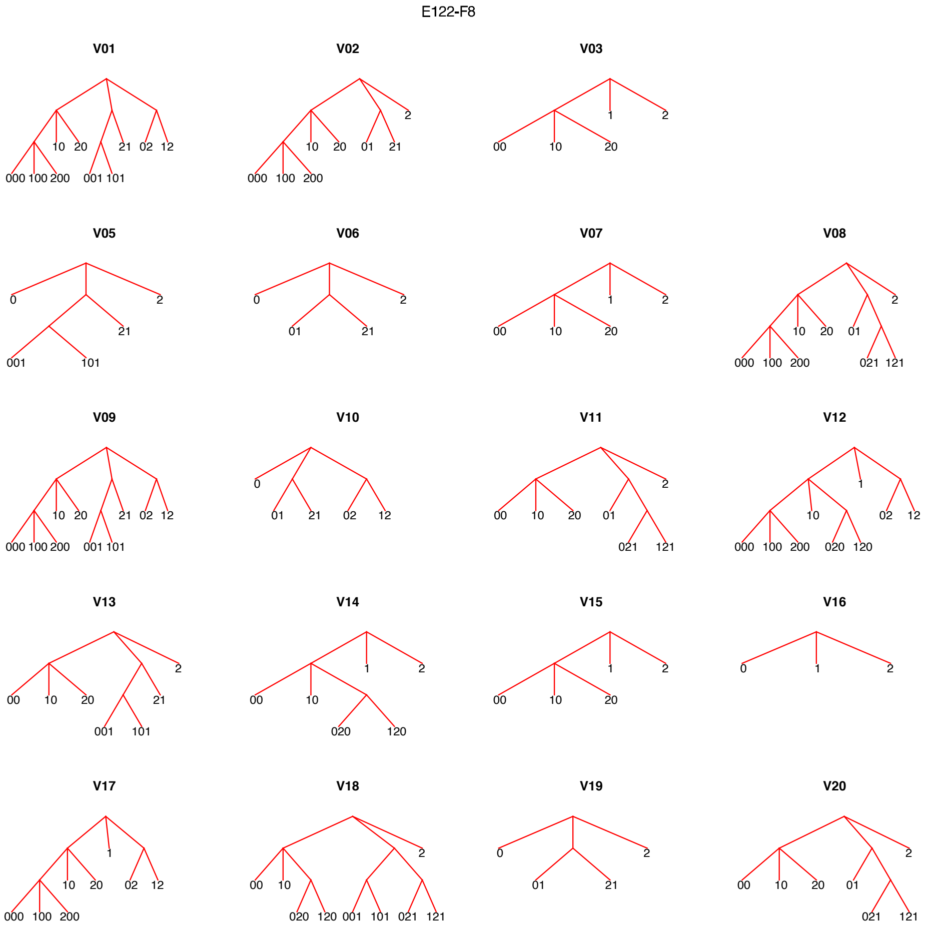

Supplementary Figure S11. Context tree estimated for each participant on electrode F8 for the Quaternary condition.

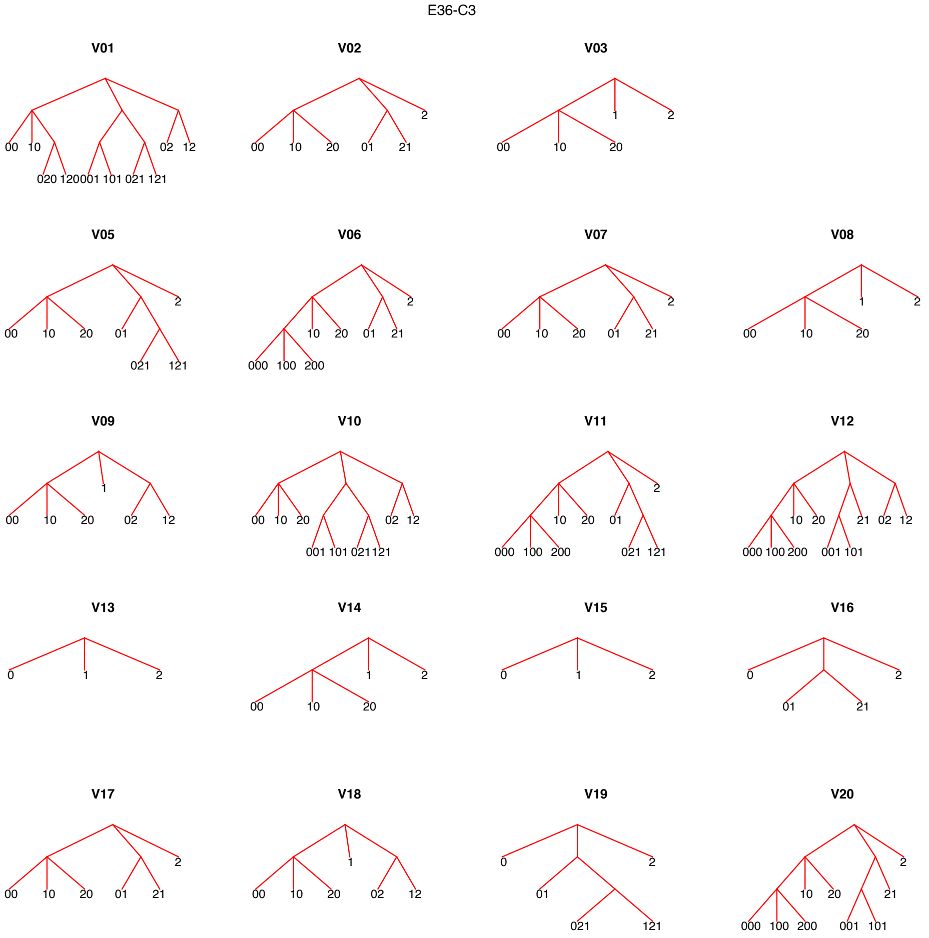

Supplementary Figure S12. Context tree estimated for each participant on electrode C3 for the Quaternary condition.

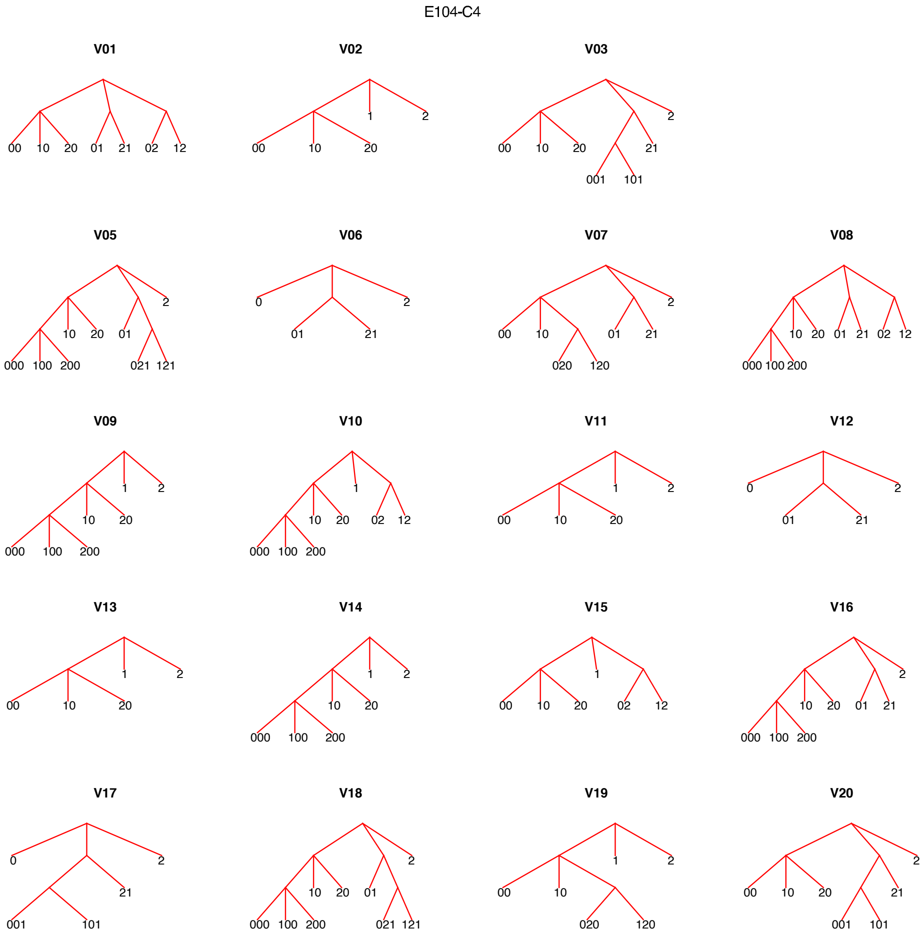

Supplementary Figure S13. Context tree estimated for each participant on electrode C4 for the Quaternary condition.

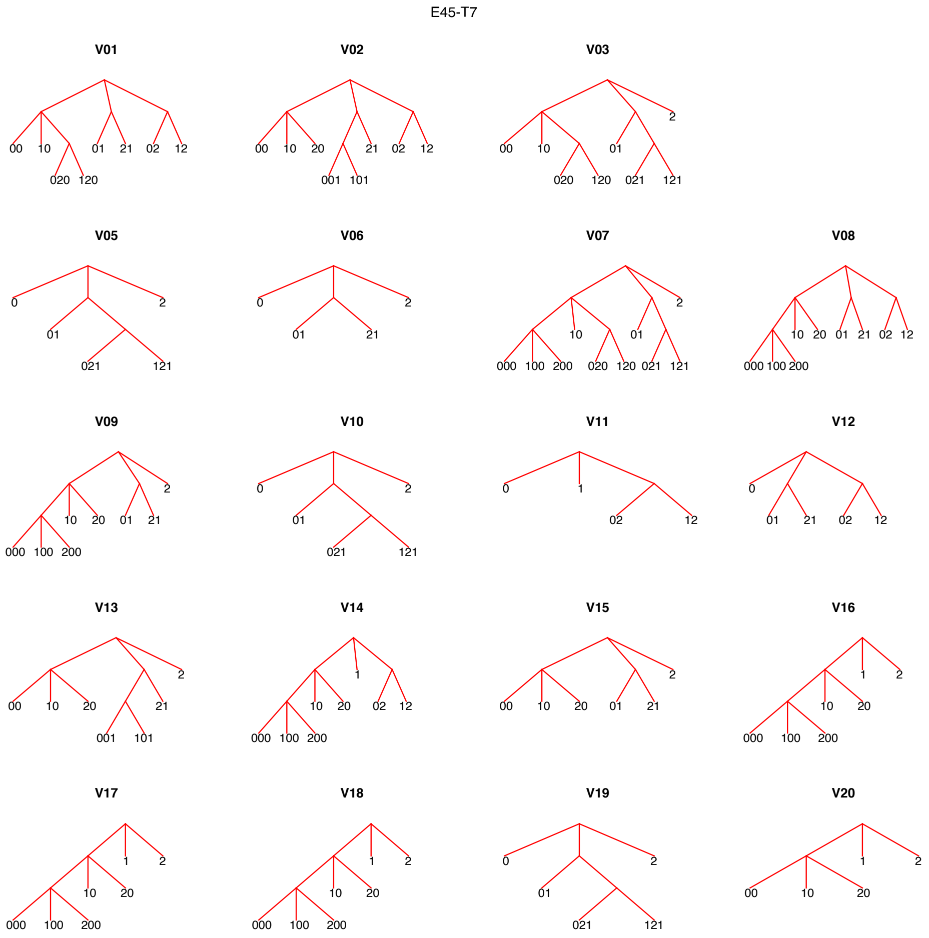

Supplementary Figure S14. Context tree estimated for each participant on electrode T7 for the Quaternary condition.

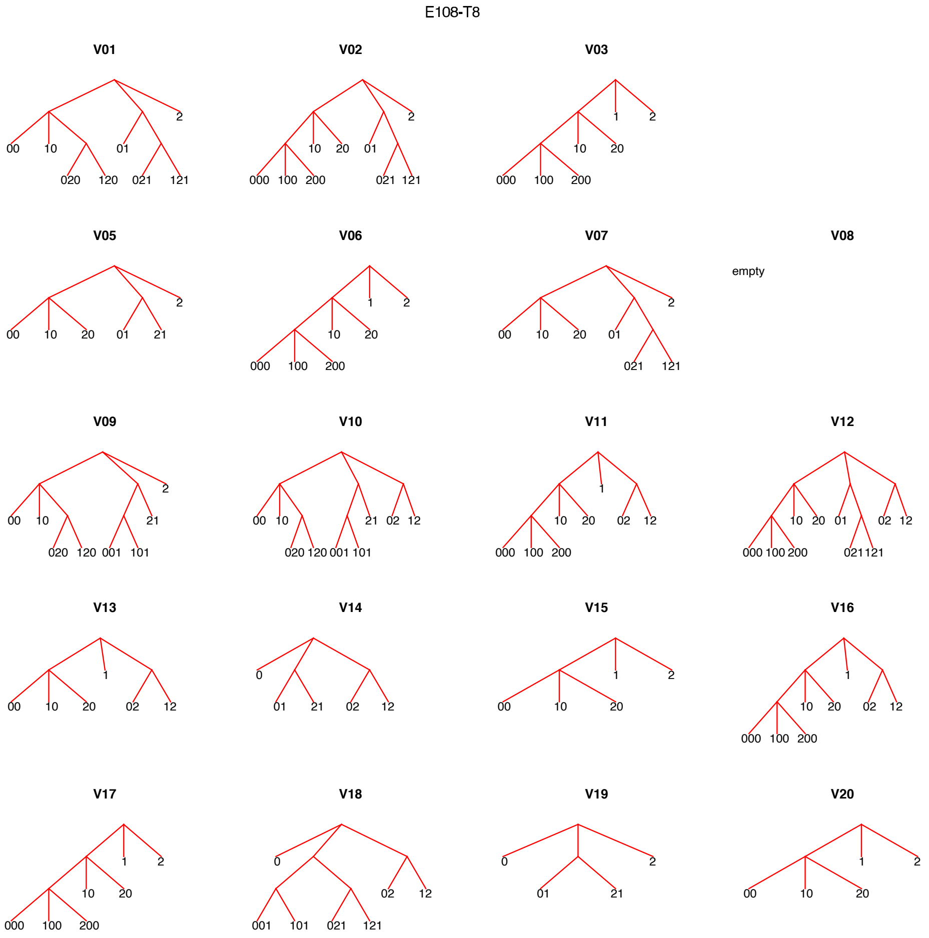

Supplementary Figure S15. Context tree estimated for each participant on electrode T8 for the Quaternary condition.

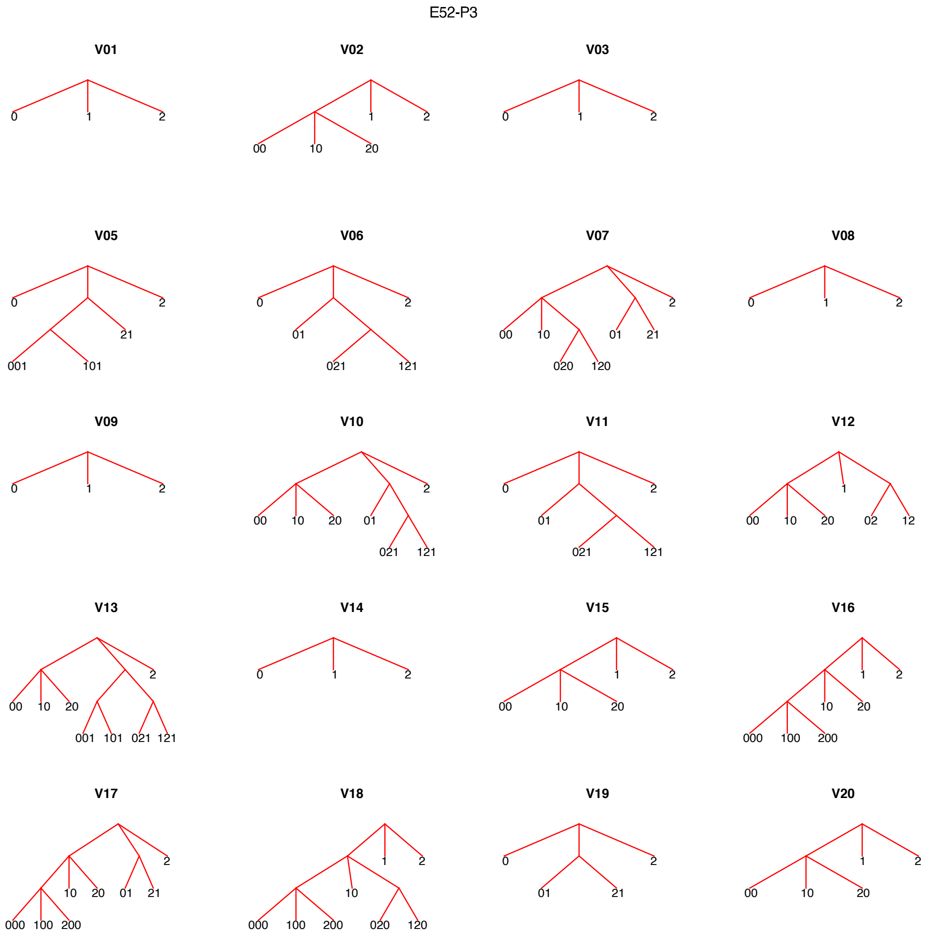

Supplementary Figure S16. Context tree estimated for each participant on electrode P3 for the Quaternary condition.

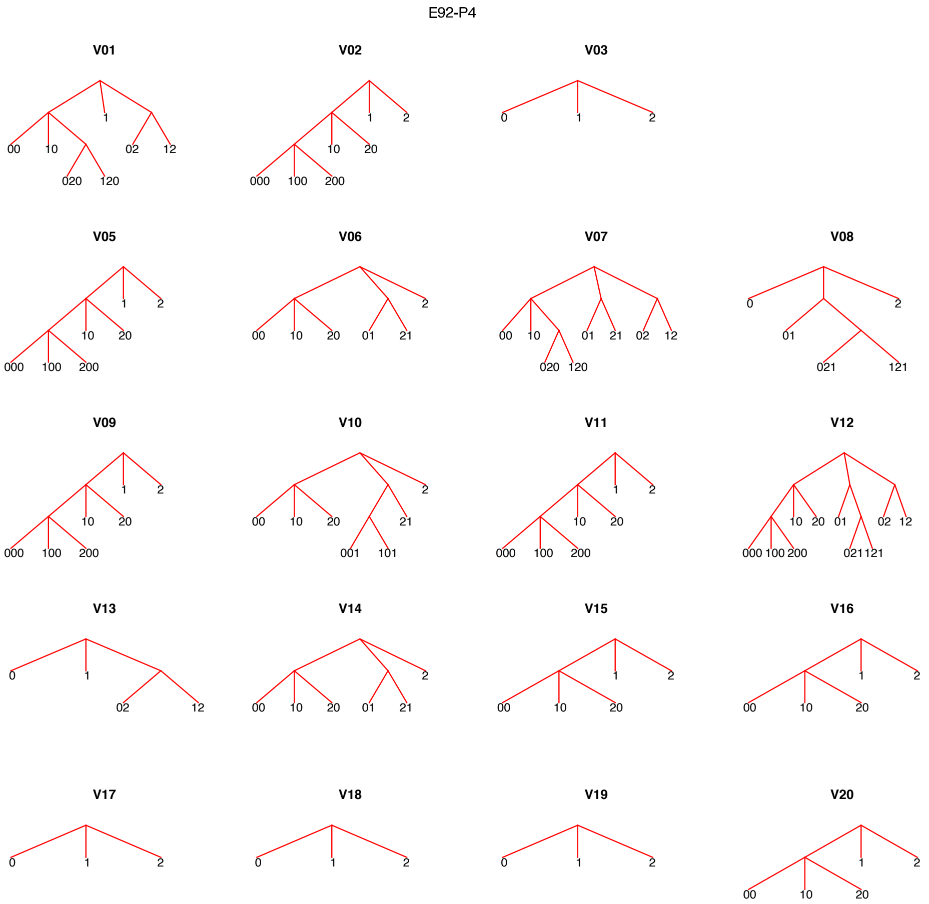

Supplementary Figure S17. Context tree estimated for each participant on electrode P4 for the Quaternary condition.

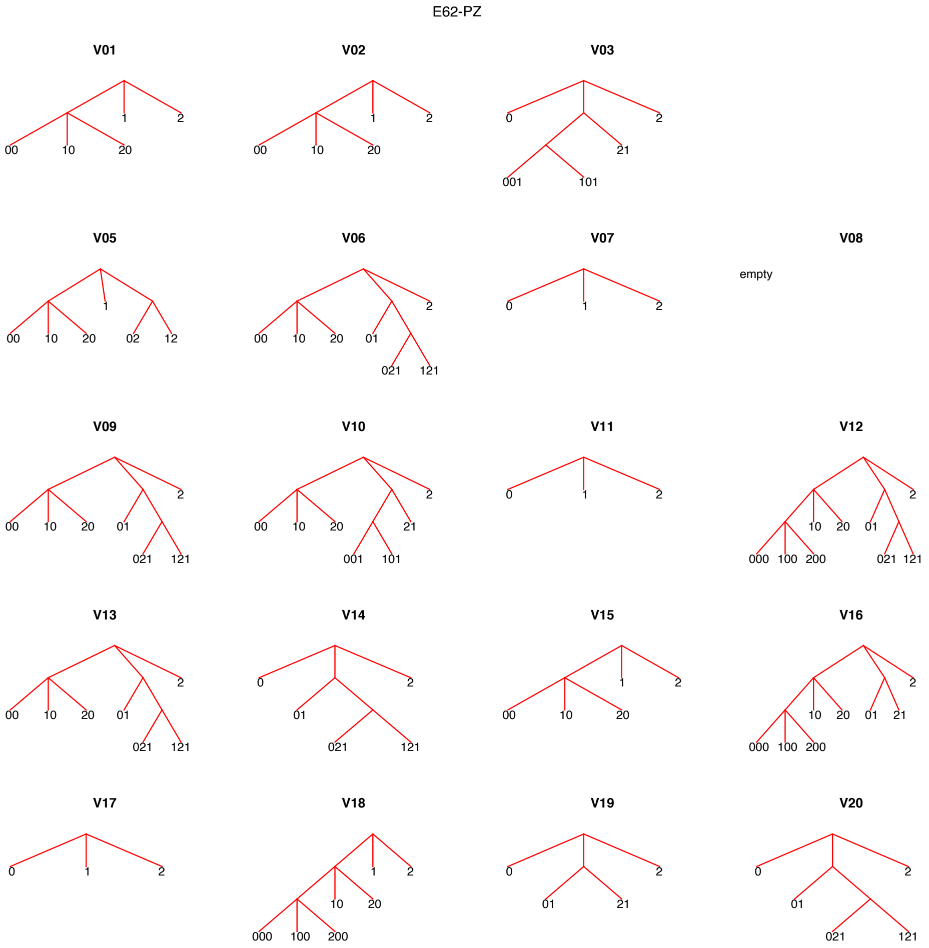

Supplementary Figure S18. Context tree estimated for each participant on electrode PZ for the Quaternary condition.

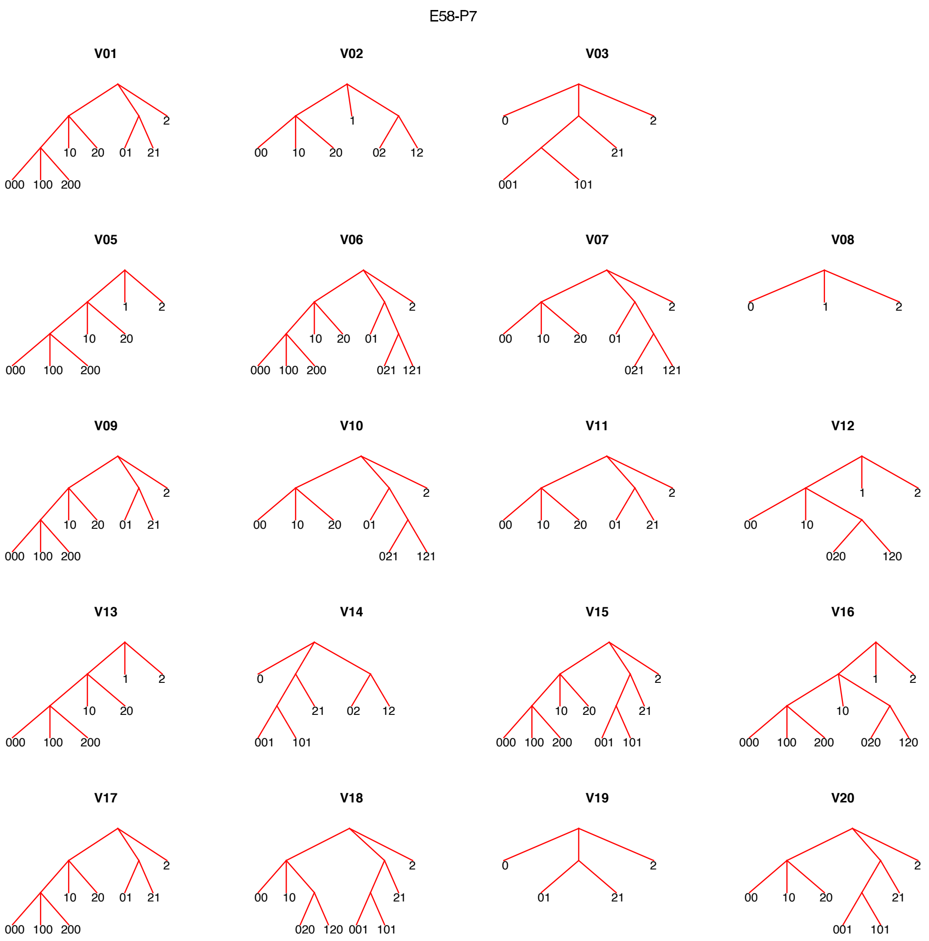

Supplementary Figure S19. Context tree estimated for each participant on electrode P7 for the Quaternary condition.

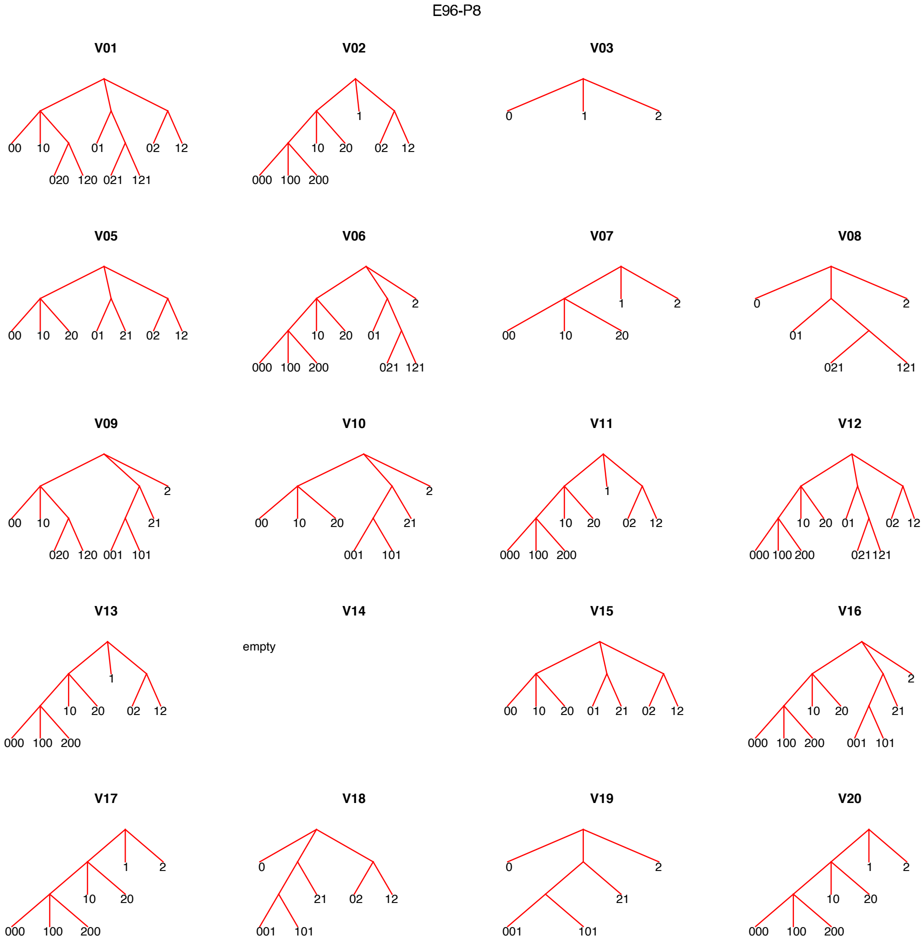

Supplementary Figure S20. Context tree estimated for each participant on electrode P8 for the Quaternary condition.

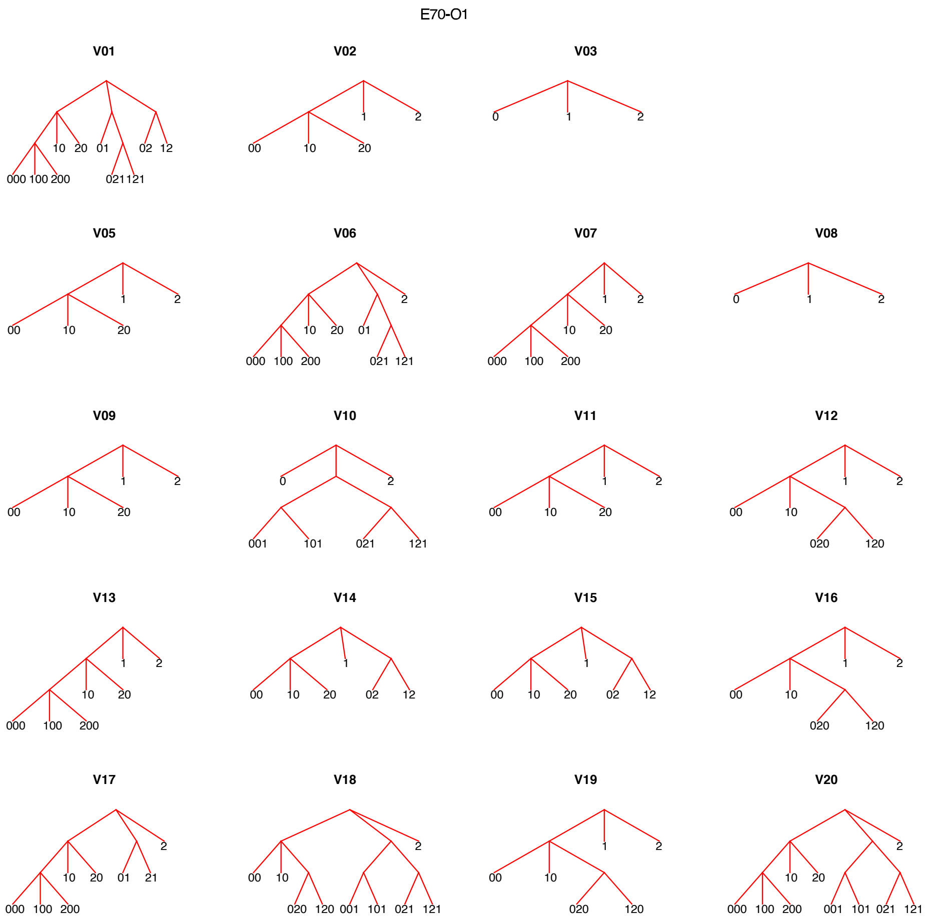

Supplementary Figure S21. Context tree estimated for each participant on electrode O1 for the Quaternary condition.

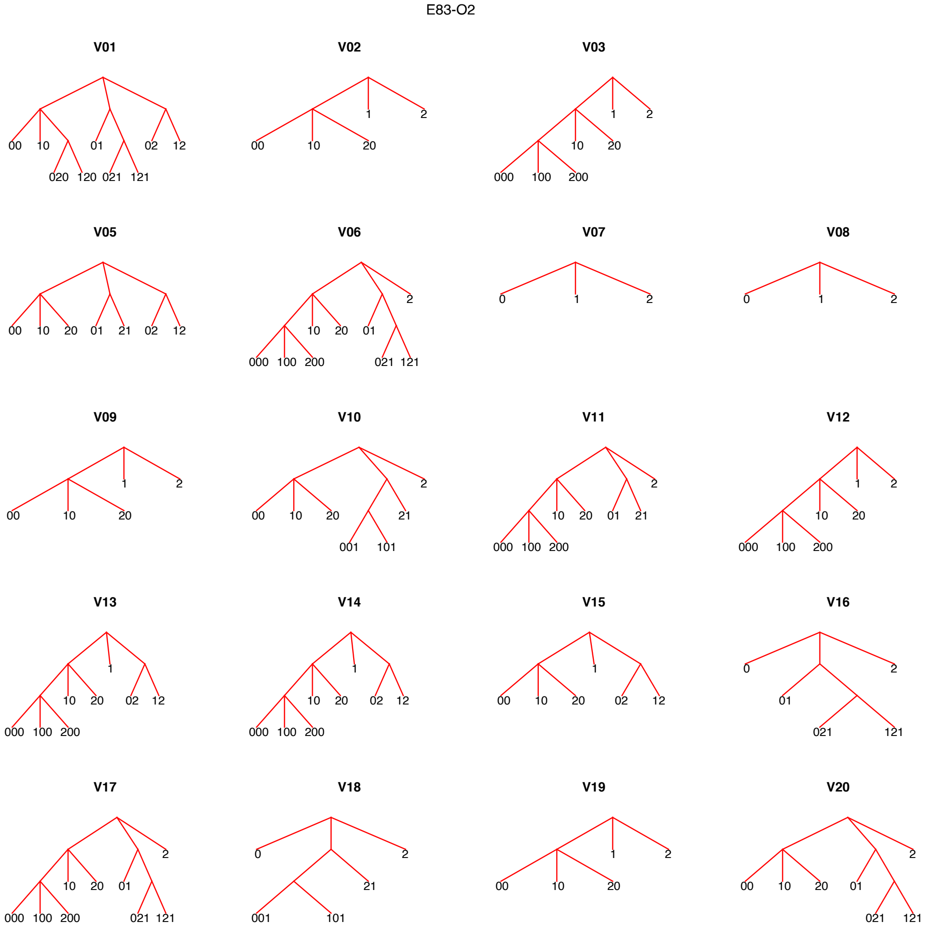

Supplementary Figure S22. Context tree estimated for each participant on electrode O2 for the Quaternary condition.

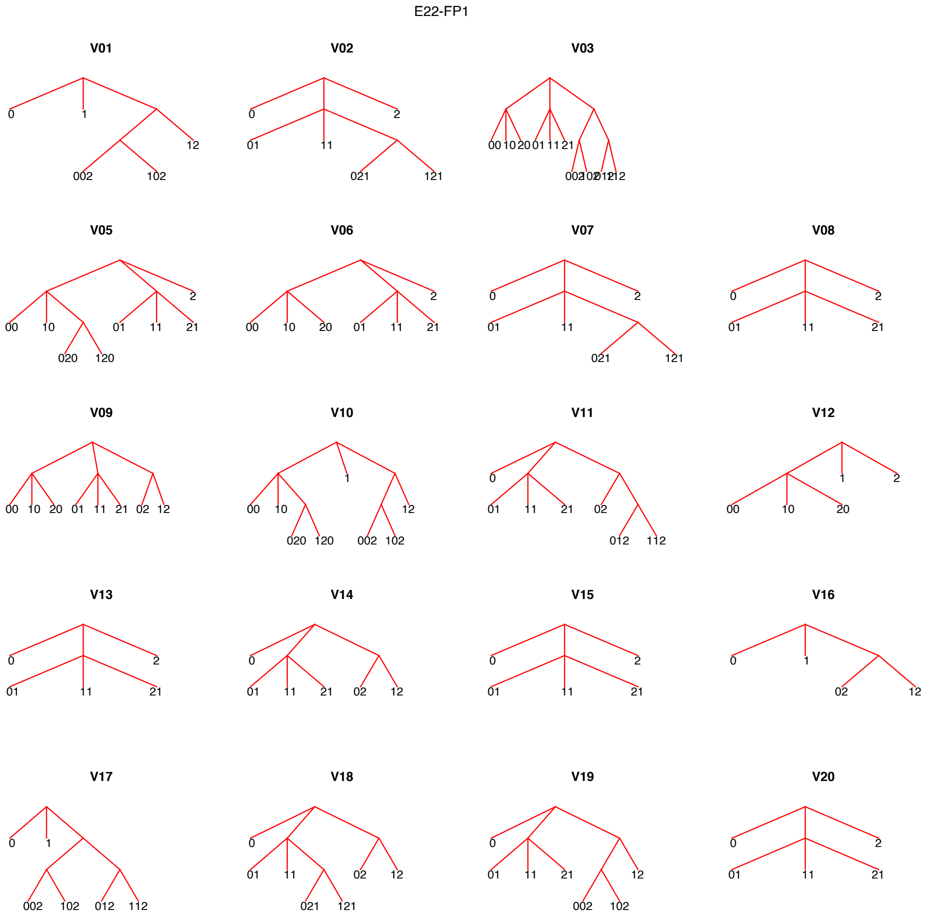

Supplementary Figure S23. Context tree estimated for each participant on electrode FP1 for the Ternary condition.

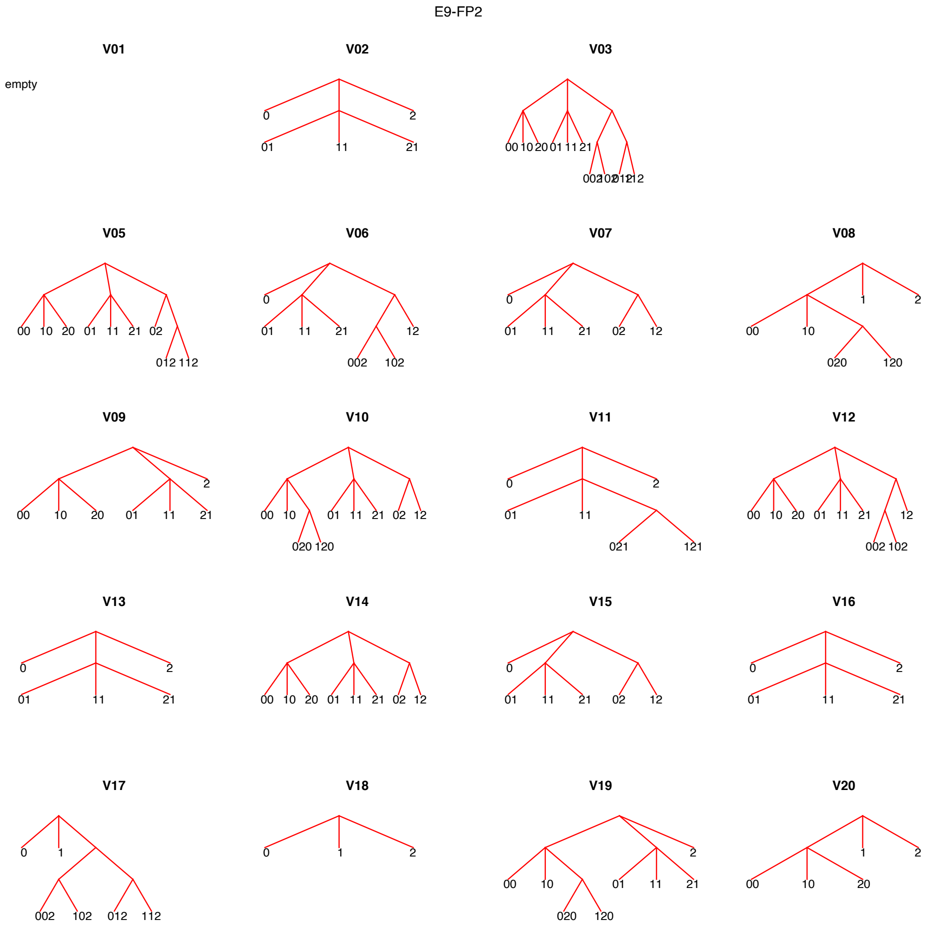

Supplementary Figure S24. Context tree estimated for each participant on electrode FP2 for the Ternary condition.

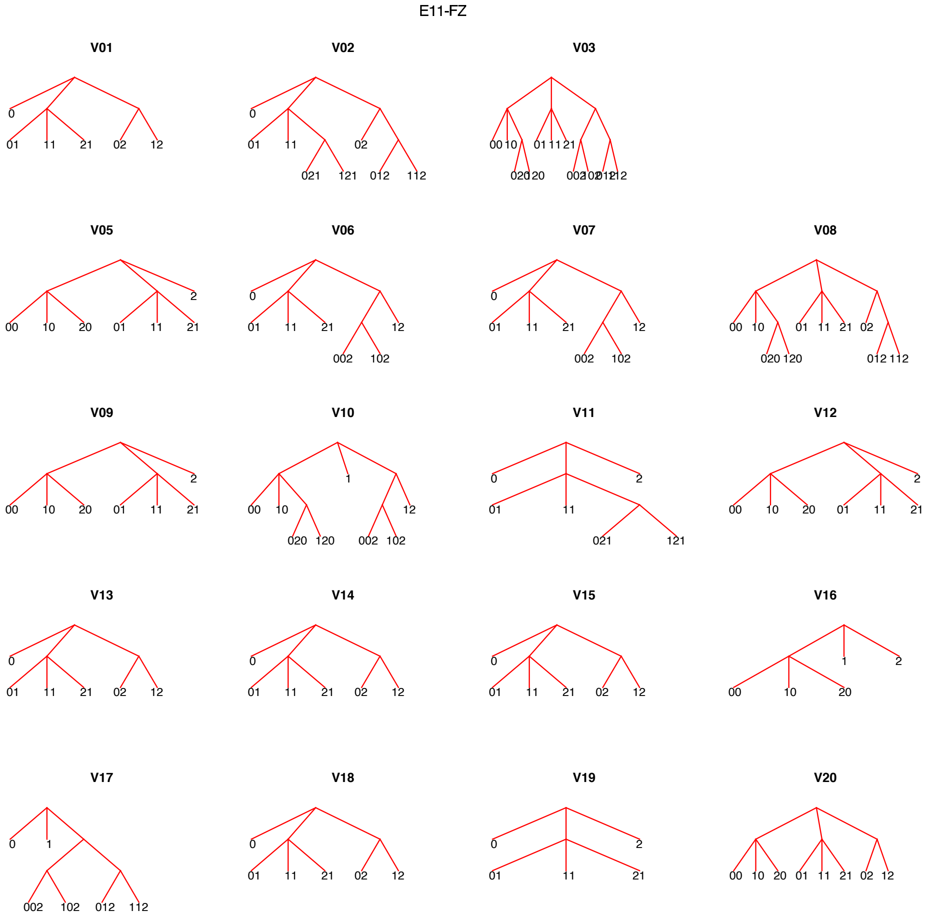

Supplementary Figure S25. Context tree estimated for each participant on electrode FZ for the Ternary condition.

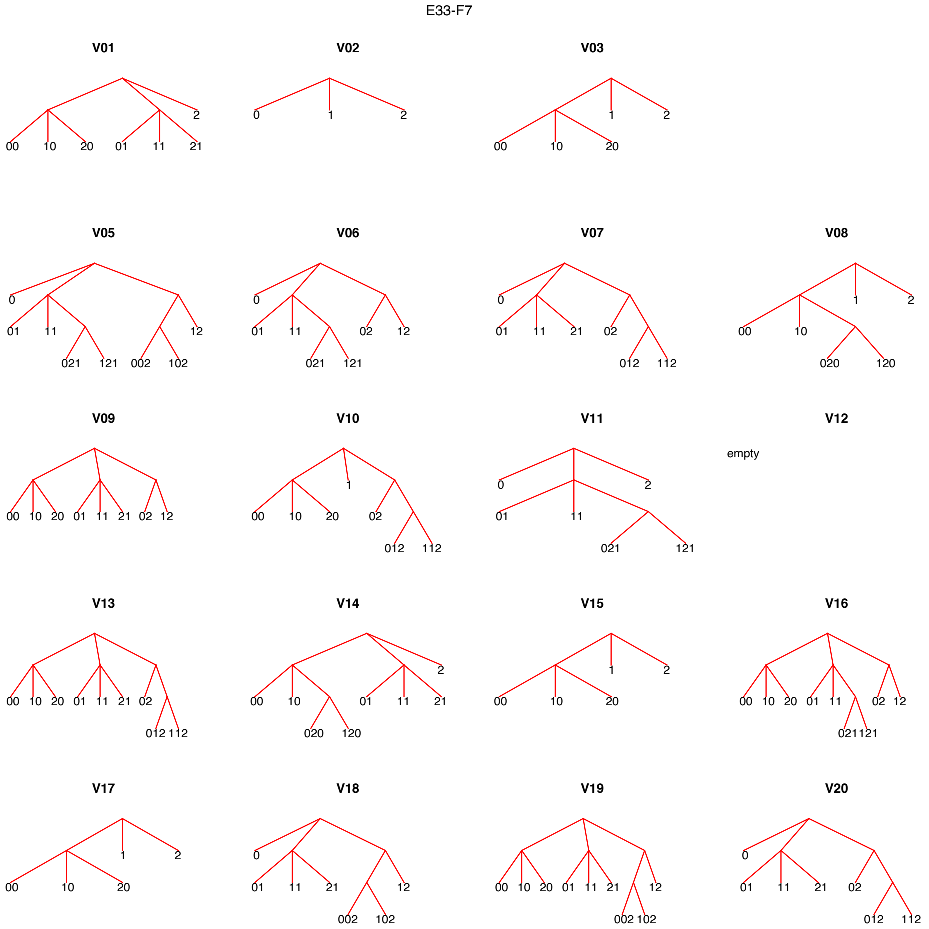

Supplementary Figure S26. Context tree estimated for each participant on electrode F7 for the Ternary condition.

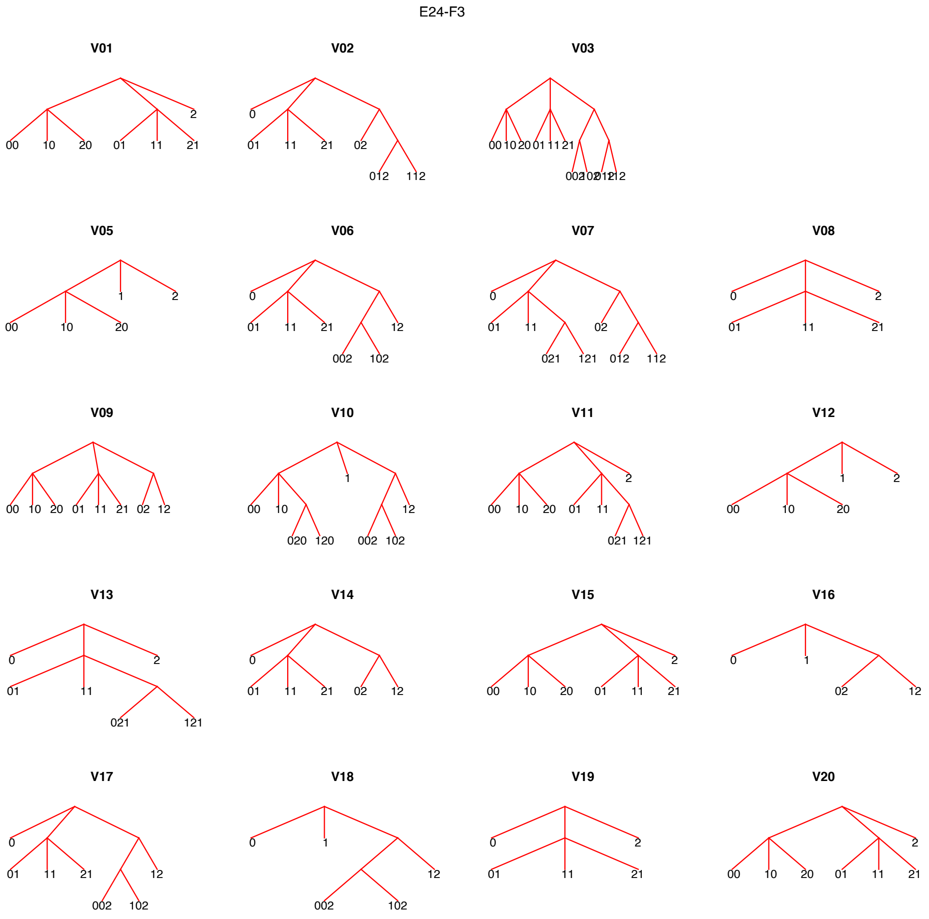

Supplementary Figure S27. Context tree estimated for each participant on electrode F3 for the Ternary condition.

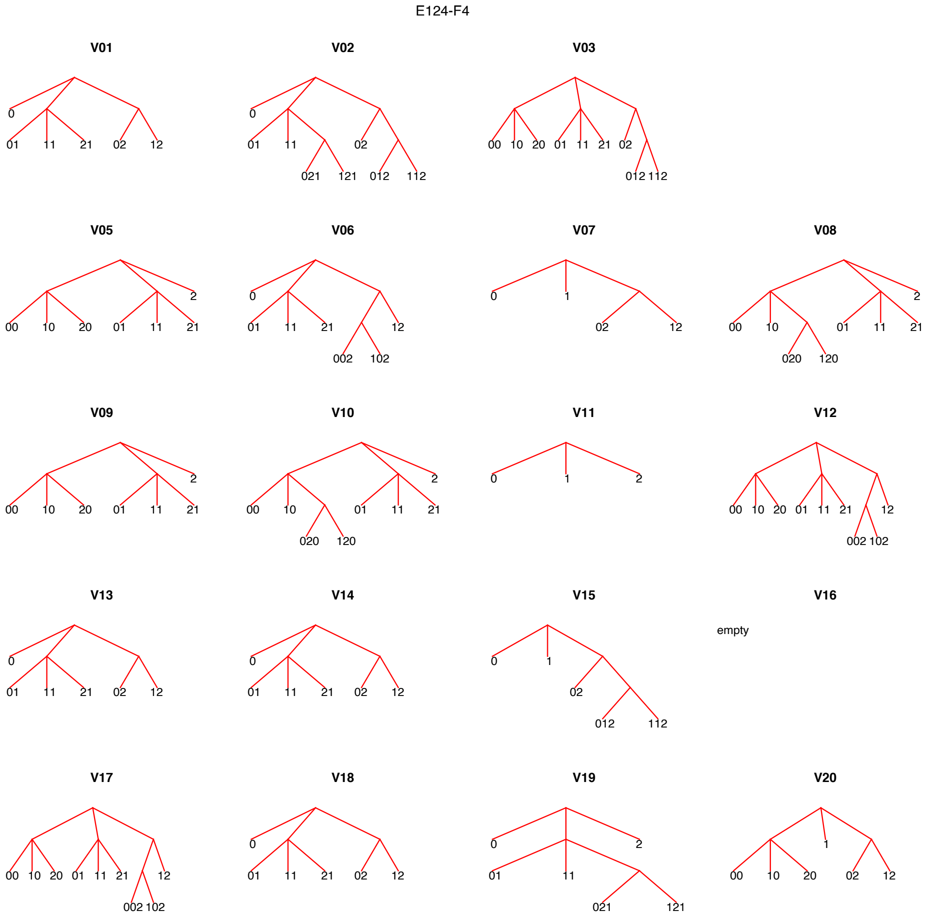

Supplementary Figure S28. Context tree estimated for each participant on electrode F4 for the Ternary condition.

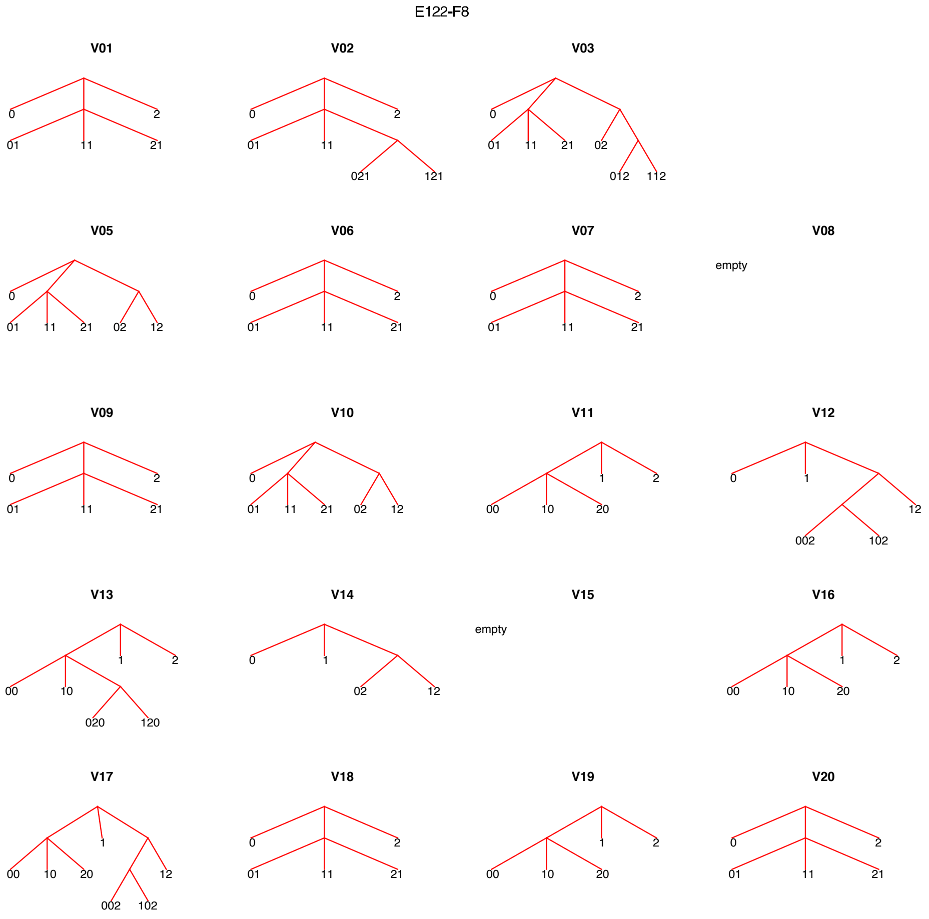

Supplementary Figure S29. Context tree estimated for each participant on electrode F8 for the Ternary condition.

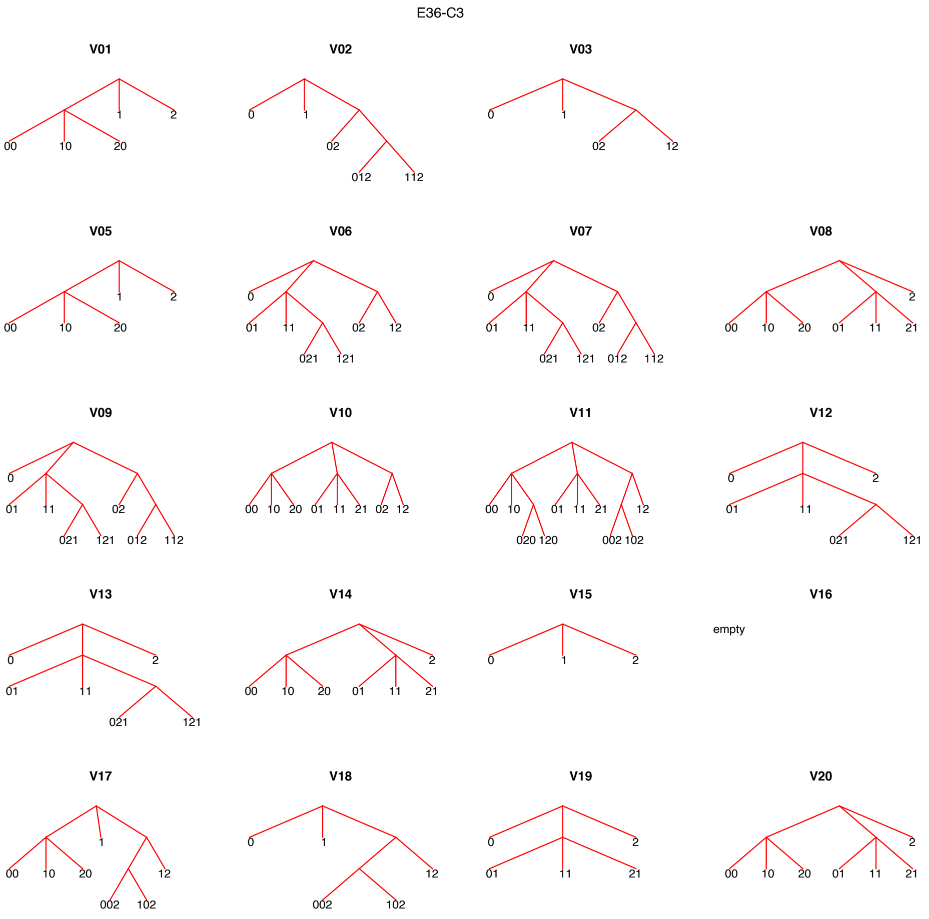

Supplementary Figure S30. Context tree estimated for each participant on electrode C3 for the Ternary condition.

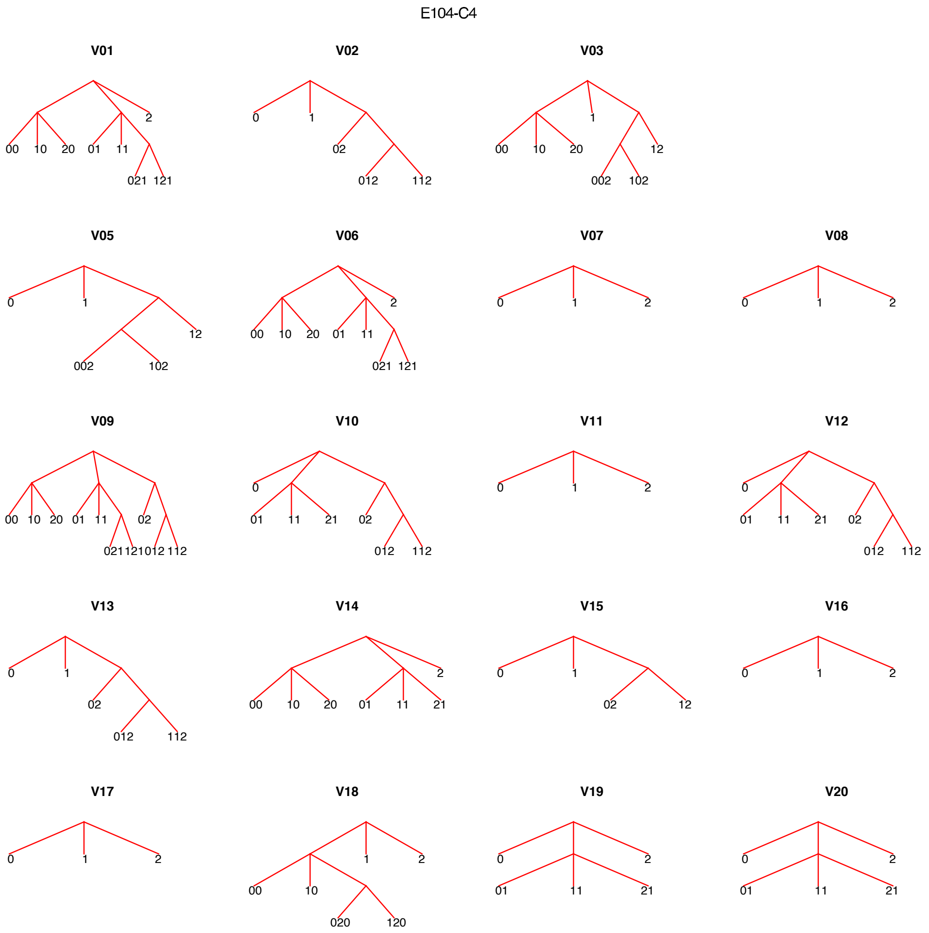

Supplementary Figure S31. Context tree estimated for each participant on electrode C4 for the Ternary condition.

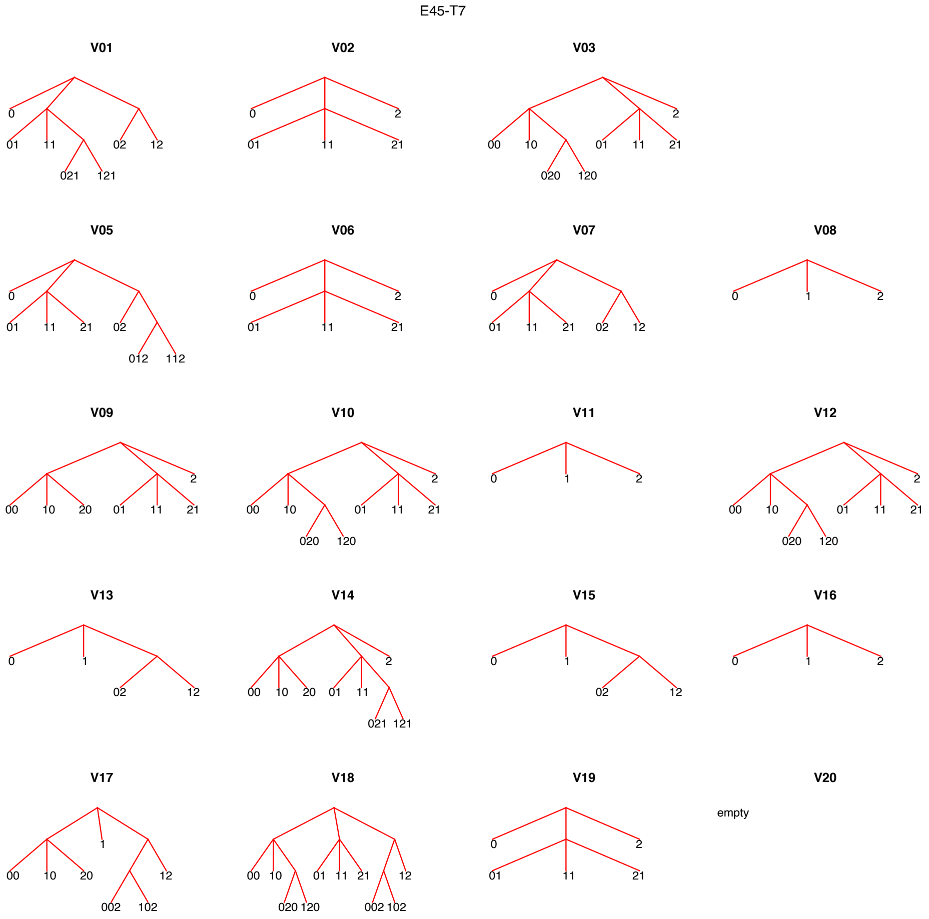

Supplementary Figure S32. Context tree estimated for each participant on electrode T7 for the Ternary condition.

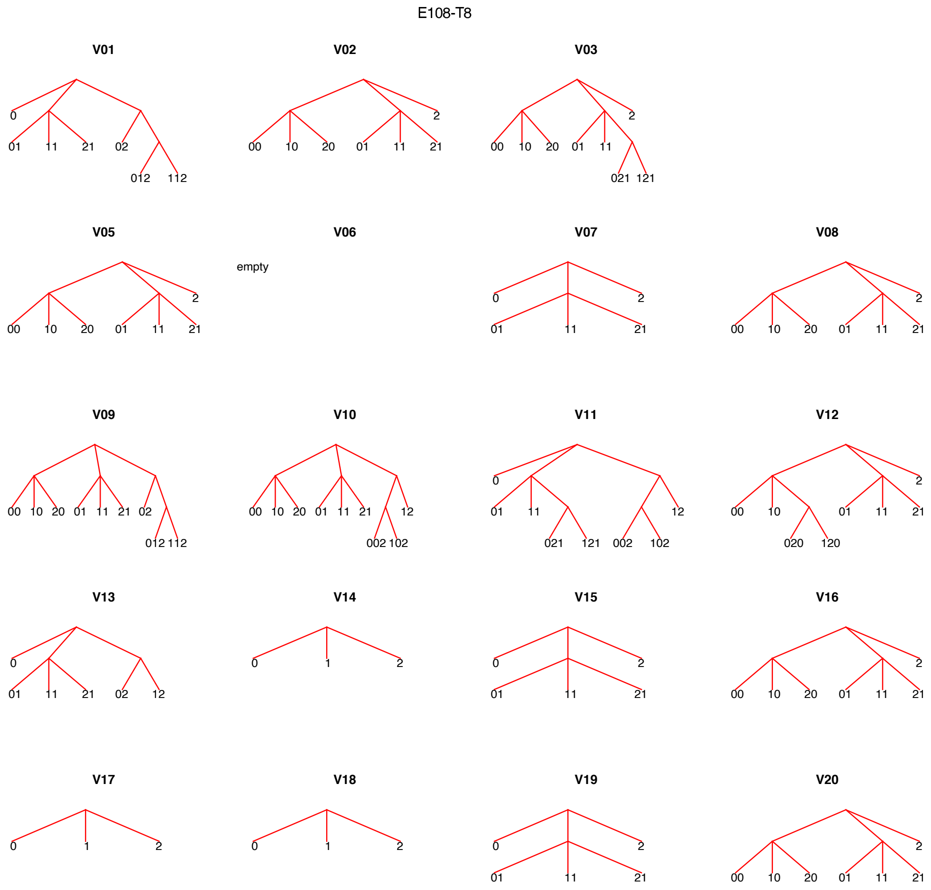

Supplementary Figure S33. Context tree estimated for each participant on electrode T8 for the Ternary condition.

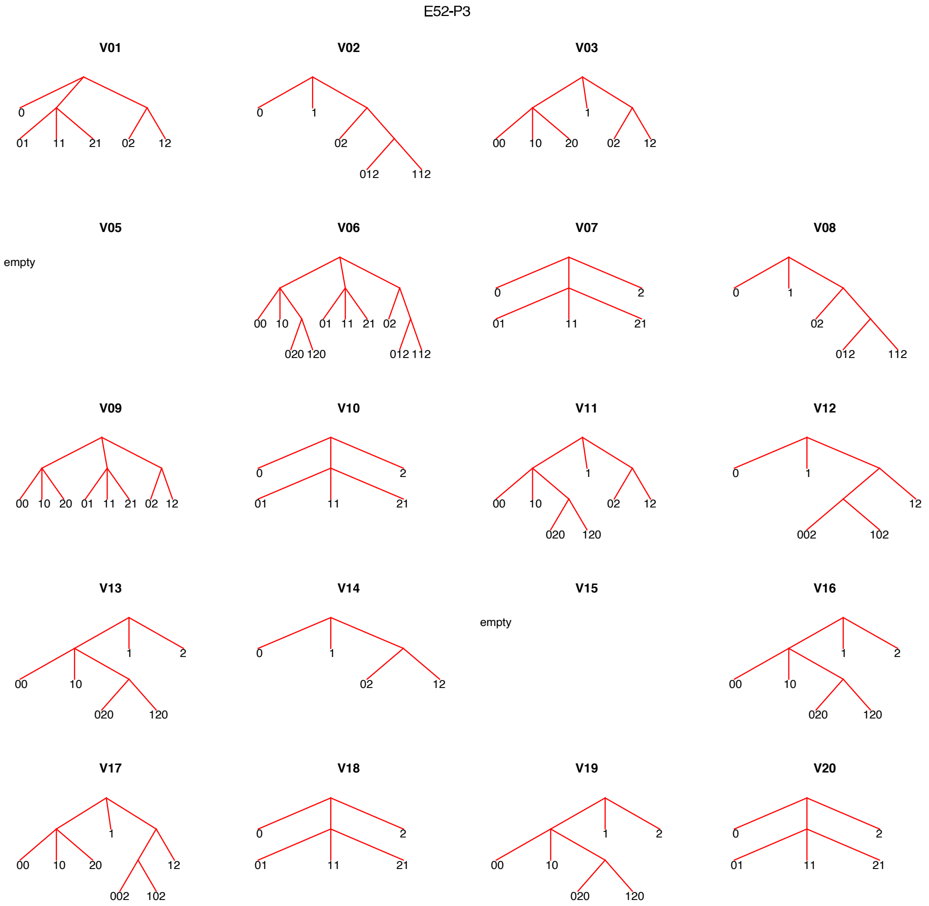

Supplementary Figure S34. Context tree estimated for each participant on electrode P3 for the Ternary condition.

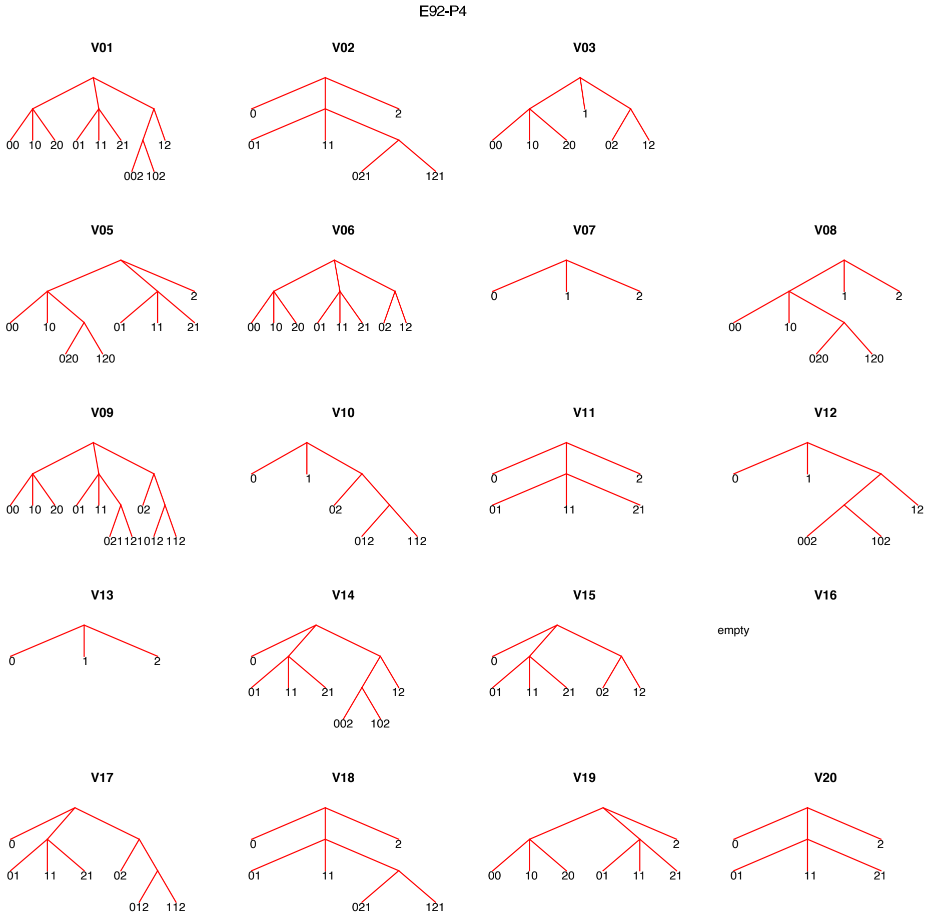

Supplementary Figure S35. Context tree estimated for each participant on electrode P4 for the Ternary condition.

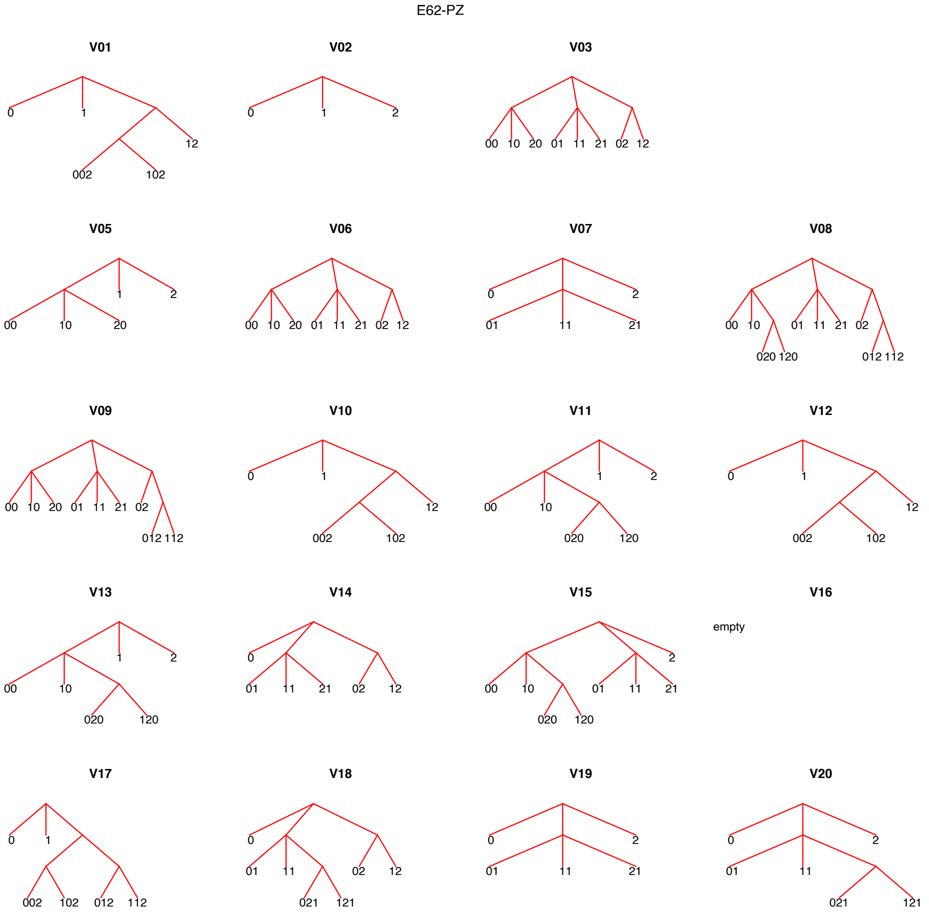

Supplementary Figure S36. Context tree estimated for each participant on electrode PZ for the Ternary condition.

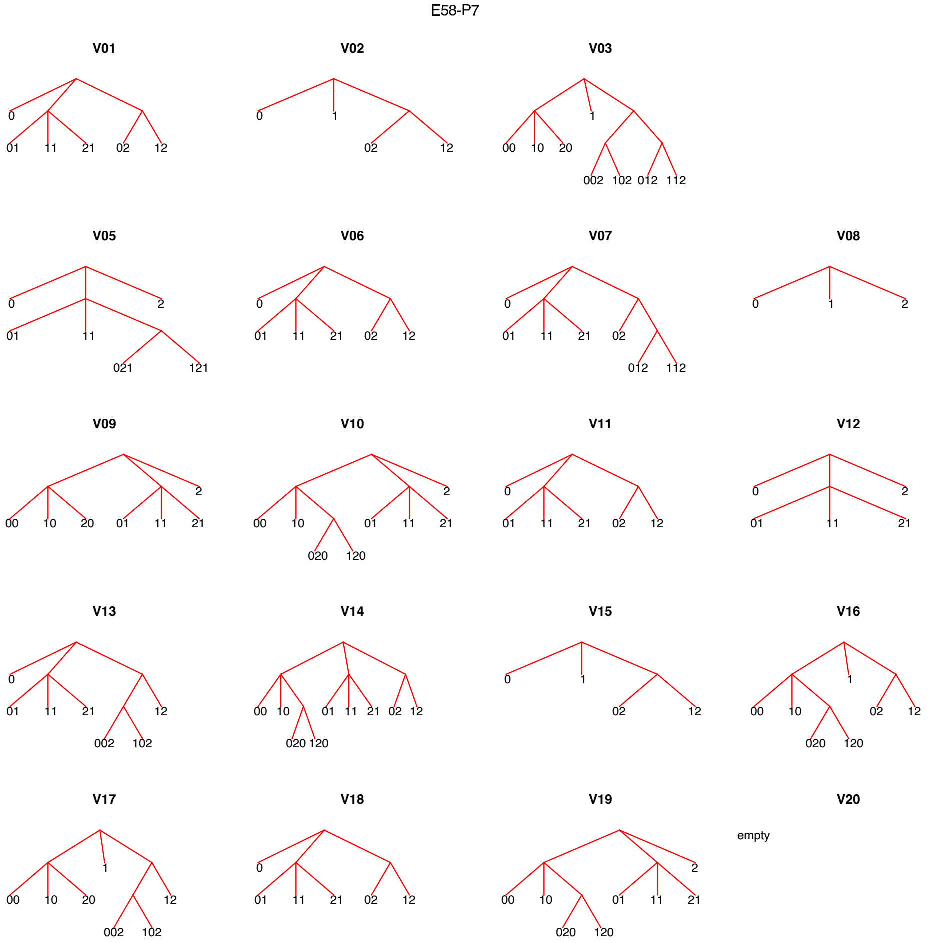

Supplementary Figure S37. Context tree estimated for each participant on electrode P7 for the Ternary condition.

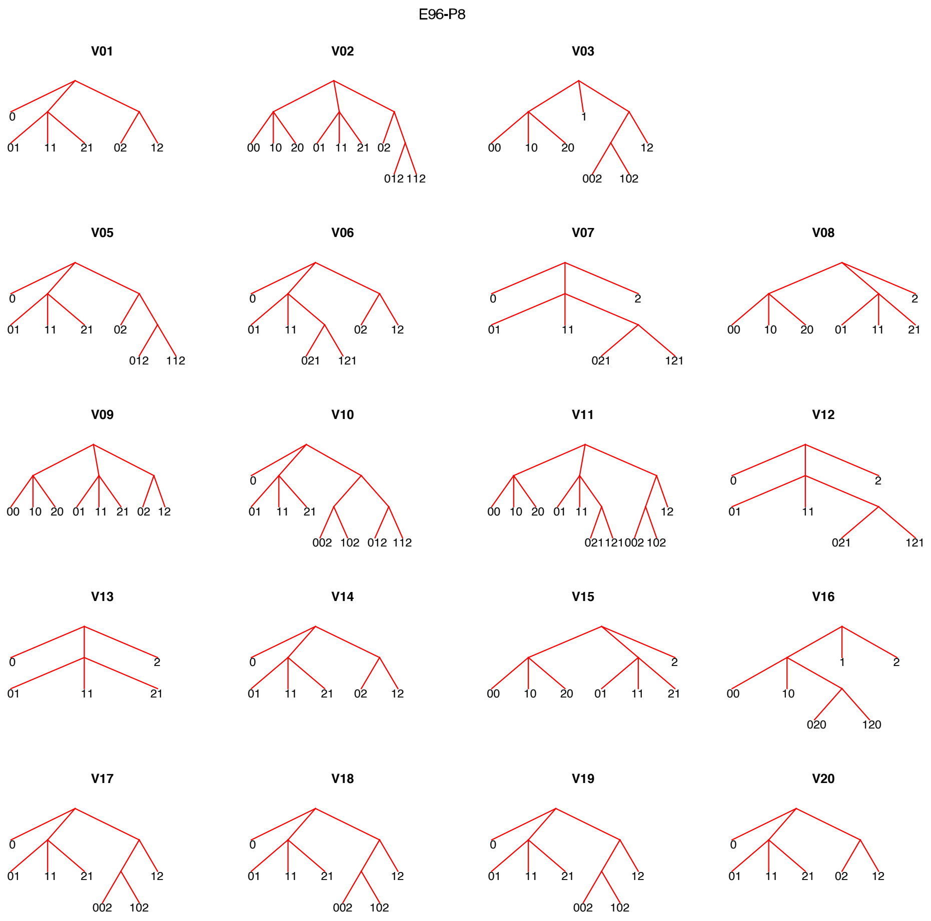

Supplementary Figure S38. Context tree estimated for each participant on electrode P8 for the Ternary condition.

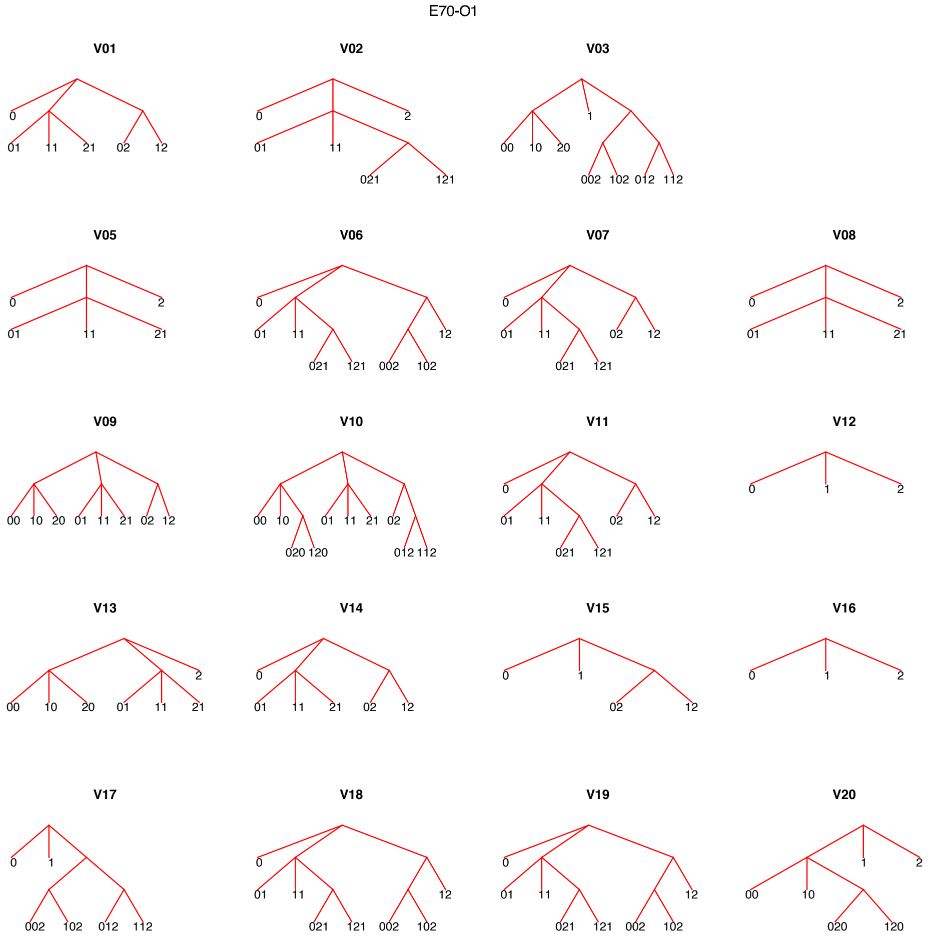

Supplementary Figure S39. Context tree estimated for each participant on electrode O1 for the Ternary condition.

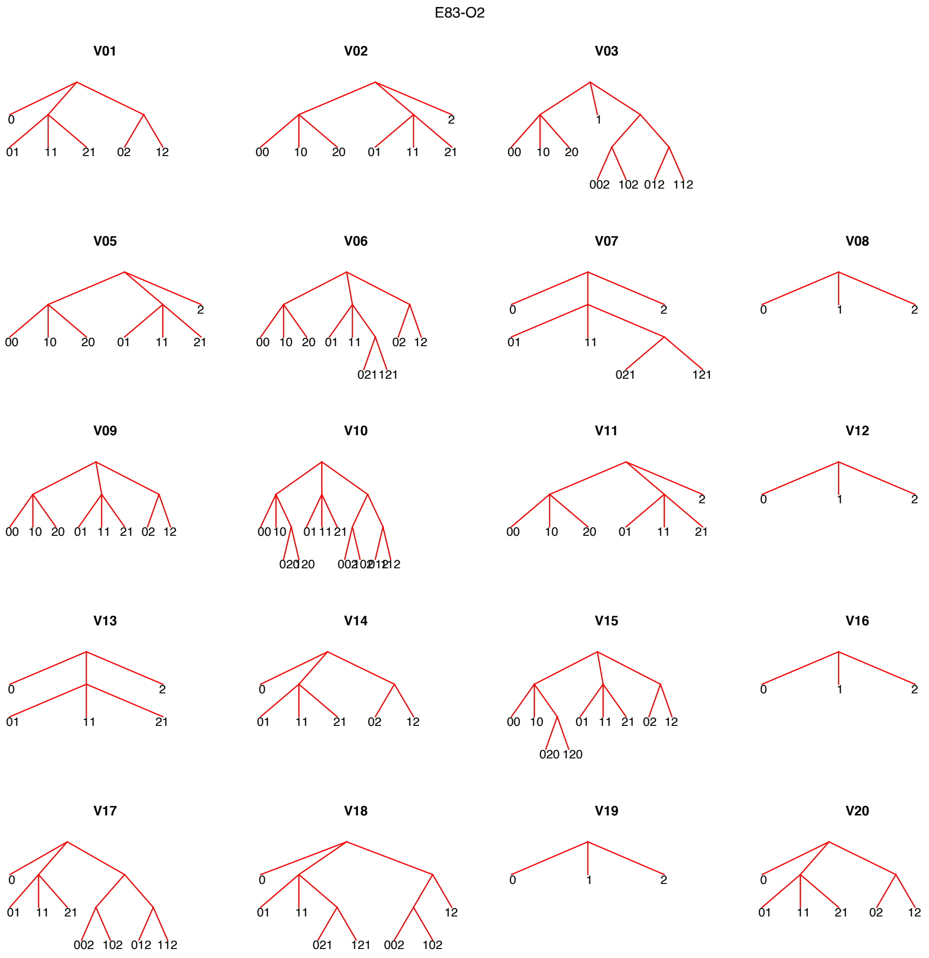

Supplementary Figure S40. Context tree estimated for each participant on electrode O2 for the Ternary condition.
